# Cascade Processes with Micellar Reaction Media: Recent Advances and Future Directions

**DOI:** 10.3390/molecules27175611

**Published:** 2022-08-31

**Authors:** Christina Tang, Bridget T. McInnes

**Affiliations:** 1 Chemical and Life Science Engineering Department, Virginia Commonwealth University, Richmond, VA 23284, USA; 2 Computer Science Department, Virginia Commonwealth University, Richmond, VA 23284, USA

**Keywords:** micelle, block copolymer, nanoreactor, one-pot, E-factor

## Abstract

Reducing the use of solvents is an important aim of green chemistry. Using micelles self-assembled from amphiphilic molecules dispersed in water (considered a green solvent) has facilitated reactions of organic compounds. When performing reactions in micelles, the hydrophobic effect can considerably accelerate apparent reaction rates, as well as enhance selectivity. Here, we review micellar reaction media and their potential role in sustainable chemical production. The focus of this review is applications of engineered amphiphilic systems for reactions (surface-active ionic liquids, designer surfactants, and block copolymers) as reaction media. Micelles are a versatile platform for performing a large array of organic chemistries using water as the bulk solvent. Building on this foundation, synthetic sequences combining several reaction steps in one pot have been developed. Telescoping multiple reactions can reduce solvent waste by limiting the volume of solvents, as well as eliminating purification processes. Thus, in particular, we review recent advances in “one-pot” multistep reactions achieved using micellar reaction media with potential applications in medicinal chemistry and agrochemistry. Photocatalyzed reactions in micellar reaction media are also discussed. In addition to the use of micelles, we emphasize the process (steps to isolate the product and reuse the catalyst).

## 1. Introduction

The annual production of organic solvents has been estimated to be nearly 20 million metric tons. Such solvents are necessary for chemical reactions. Furthermore, to achieve sufficient product purity, large excesses of solvent are necessary for extractions, purifications, and cleaning processes. In particular, fine chemical and pharmaceutical manufacturing requires large amounts of solvent. To prepare active pharmaceutical ingredients, solvent can account for up to 85% of the mass handled, which generates large amounts of hazardous waste per mass of desired product (quantified by E factors presented in Table 1). Such consumption of organic solvents is considered unstainable [1,2].

Reducing (or eliminating) the use of solvents is an important aim of green chemistry. Several classes of solvents have been suggested as ‘green’ solvents, including supercritical fluids, ionic liquids, and solvents derived from biomass. Reviews of such solvent classes are available elsewhere [1]. Solvent selection can affect reaction yield, waste, and operating conditions. Thus, it is important to consider the solvent, as well as the process (including potential solvent recovery, e.g., by distillation or nanofiltration) [4]. Water is also considered a green solvent, as it is non-toxic. Using micelles self-assembled from amphiphilic molecules dispersed in water has facilitated reactions of organic compounds with water as the bulk solvent [5,6]. Furthermore, the hydrophobic effect can be leveraged to considerably accelerate the apparent reaction rate, as well as enhance selectivity [7]. Thus, micellar reaction media is a promising approach to reduce the use of solvents in organic chemistry. 

Here, we review micellar reaction media and their potential role in sustainable chemical production. The focus of this review is the application of engineered amphiphilic systems (namely, surface-active ionic liquids, designer surfactants, and block copolymers) for reactions. In particular, we emphasize “one-pot” multistep reactions achieved using micellar reaction media. Photocatalyzed reactions are also discussed. In addition to the reactions achieved using micelles, we emphasize the process (steps to isolate the product and reuse the catalyst). Green chemistry metrics to assess and compare processes are also briefly discussed. 

## 2. Background

### 2.1. Micelle Fundamentals 

**Figure 1:**
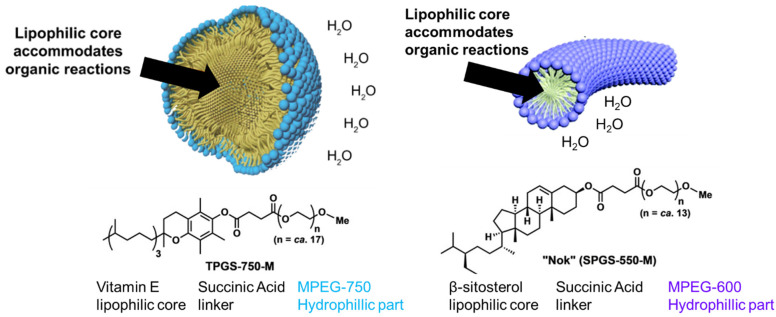
Schematics of self-assembled micelles from amphiphilic molecules for organic reactions. The designer amphiphilic surfactant TPGS-750-M self-assembles into spherical micelles dispersed in water. The hydrophobic core (composed of vitamin E) accommodates organic reactions using water as the bulk solvent. The designer amphiphilic surfactant “Nok” self-assembles into cylindrical structures (rod-like micelles) dispersed in water. The hydrophobic core (composed of β-sitosterol) accommodates reactions using water as the bulk solvent. Adapted with permission from [11,12].

Micellar reaction media comprise amphiphilic molecules (surfactants and block copolymers) dispersed in water (bulk solvent). In water, above a critical micelle concentration (CMC) [8], amphiphilic molecules spontaneously self-assemble into spherical micelle structures with a lipophilic core (shielded from the water) and hydrophilic shell (exposed to the water) due to the hydrophobic effect. The hydrophobic core is effectively a small volume of liquid hydrocarbon with the ability to dissolve hydrophobic substances in order to accommodate chemical reactions. For conventional surfactant micelles, nanoscale assemblies form due to aggregation of 50–100 surfactant molecules in thermodynamic equilibrium. At equilibrium, there is constant exchange of the individual amphiphilic surfactant molecules among aggregates. The typical lifetime of a surfactant micelle is on the order of milliseconds [7]. The morphology of the aggregate that forms depends on amphiphile concentration and the size of the hydrophobic portion of the amphiphile relative to that of the hydrophilic portion, as well as experimental conditions, such as temperature, pH, and ionic strength [7]. Medium aggregates (i.e., micelles 50 to 60 nm in diameter) can improve apparent reaction performance compared to other sizes (10 to 15 nm or larger than 150 nm) [9]. For some amphiphilic molecules, a transition from sphere- to rod-shaped (cylindrical) micelles can be observed with increasing amphiphile concentration (Figure 1) [10]. 

Overall, micellar media are microheterogenous and provide a variety of solubilization environments ranging from a “hydrocarbon-like” core to bulk water. This microheterogenous structure provides unique microenvironments for different molecule, as well as distinct parts of the same molecule. Micelles isolate species from the bulk solvent, improving solubilization of organic reagents in water, increasing local concentration of reactants, improving reactivity, and affecting selectivity [2,7,13]. Organic species added in the reaction media are distributed between bulk water and micelles, depending on their polarity, charge, and dimensions. Apolar substrates are almost exclusively hosted within the micelles, resulting in high local concentration. Multicomponent reactions are promoted by confinement (spatial restriction of reactants, intermediates, and catalysts within the structure of nanoscale dimensions) [14]. Confinement also results in unique adsorption behavior, in addition to increased local concentration of the reactants [13]. Reaction pathways and rates can also be affected by the location and orientation of the solubilized species within micelles, as well as the degree of electrostatic stabilization of the transition state [7,15]. More in-depth discussions of the mechanisms of reaction (e.g., “in water” and “on water” surfactant–catalyst interactions) are available elsewhere [16,17]. We also note that multiphase systems, such as emulsions [18] and microemulsions [19], are outside the scope of this review. In this work, we briefly highlight the benefits of micellar reaction media with respect to the performance of organic reactions performed using water as the bulk solvent (single phase) using some illustrative examples. 

### 2.2. Effect of Surfactant on Reaction Mechanism

The presence of surfactants can affect the reaction mechanism. For example, micelles of peptide surfactants containing histidyl residues have been used for hydrolysis of *p*-nitrophenyl carboxylates. Typically, imidazole-catalyzed hydrolysis of *p*-nitrophenyl carboxylates has occurred via a nucleophilic mechanism. In the micellar system, both general base and nucleophilic mechanisms are present. The general base mechanism is predominant, whereas the nucleophilic mechanism is suppressed due to reduced contact between the catalyst and substrate due to steric hindrance. The observed mechanism is similar to the reaction mechanism proposed for serine proteases [20]. 

Nickel complexes have been used with cationic surfactant cetyltrimethyl ammonium bromide (CTAB). Kinetic analysis was performed using the formation of Ruhemann’s purple. Reaction kinetics were affected by the concentration of the reactants in the micelle phase, which was affected by hydrophobic and electrostatic effects. The micellar phase also affected the quantity of the intermediates. Overall, the analysis suggested that the surfactant had a co-operative effect of the formation of the imine intermediate [21]. A similar co-operative effect of the surfactant on the intermediate has been observed between CTAB and *p*-nitroacetophenone (detected via NMR studies) in the Morita-Baylis-Hillman reaction of ketones [22]. More exhaustive discussions of the effect that micelles can have on the reaction mechanism are available elsewhere [19]. 

### 2.3. Effect of Surfactant on Apparent Reaction Rate

#### 2.3.1. Effect of Surfactant Charge

Performing reactions in micelles can affect both the apparent reaction rate and effective concentration of the reactant. Surfactant properties, such as charge, considerably influence reaction efficiency [23]. To date, the effect of surfactant type has been considered to be system-dependent and largely determined experimentally. For example, studying the kinetics of alkaline hydrolysis of Fe(II) complexes in micellar media, nonionic surfactant Brij 35 was found to inhibit reaction kinetics. This effect was attributed to the low affinity between the hydroxide ion and hydrophobic micelle psuedophase [24]. In another example of oxidation of d,l-aspartic acid using gold (III) examined using various surfactants, CTAB caused precipitation. Sodium dodecyl sulfate (SDS) did not affect the rate. Triton X-100 did not affect the reaction mechanism but retarded the reaction rate [25]. In contrast, anionic surfactants were used with chiral Pd catalysts to achieve C-H functionalization of indoles, whereas the catalysts were ineffective with cationic and nonionic surfactants [26]. In another example, whereas the oxidation of citric acid by permanganate was inhibited by SDS, crocin (a non-ionic sugar-based surfactant) micelles accelerated the rate by 60-fold [27,28]. 

In many examples, the use of micellar reaction media has accelerated apparent reaction rates. For example, in the alkanolysis of ionized phenyl salicylate, the apparent rate constant nearly doubled in the presence of SDS. The conversion of the reactant also increased with surfactant concentration [29]. Rate enhancement was also observed for hydration of 1,3-dichloroacetone performed in the presence of Triton X-100 micelles catalyzed by hydrochloric acid or imidazole. As the surfactant concentration increased, the effective rate constants for hydration increased. Thermodynamic analysis and kinetic analysis indicated that rate enhancement was due to the participation of the surfactant as an acid-base catalyst, as well as to changes in the structures of the transition state in the micelle compared to the reaction performed in solvent [30]. In another example, the alkaline hydrolysis of methyl decanoate was performed in the presence of various surfactants. Due to the hydrophobicity of the reactant, the reaction was relatively slow using water as the solvent. Use of nonionic surfactant (*tert*-octylphenoxy) polyethoxyethanol (TX-100) accelerated the rate of reaction by about 100-fold. This rate enhancement was attributed to enhanced reactant solubility. Using a cationic surfactant, further rate enhancement was observed due to electrostatic interactions between the substrate and surfactant [31]. 

The catalytic hydrolysis of Bis(4-nitrophenyl)phosphate (BNPP) catalyzed by α-nucleophiles was accelerated 107-fold in the presence of cationic Gemini surfactants. The rate enhancement was attributed to increased nucleophilic reactivity of HOO- in the Gemini surfactant micelles [32]. In contrast, Diels-Alder reactions of dienophiles with cyclopentadiene in aqueous media were accelerated by anionic surfactants, such as SDS (70-fold rate enhancement compared to the uncatalyzed reaction) and linear alkylbenzene sulfonic acid (170-fold rate enhancement compared to the uncatalyzed reaction). The reaction was inhibited by cationic surfactants. These results were attributed to the interactions of the acid catalyst with the micelle surface [23].

#### 2.3.2. Effect of Surfactant Concentration

Effective reaction rates are also affected by micelle size and shape, which are influenced by surfactant concentration. For example, organophosphates (soman and sarin) have been hydrolyzed by *o*-iodosobenzoic acid in the presence of aqueous micellar cetyltrimethylammonium chloride (CTAC) [33]. The observed rate constant increased with surfactant concentration. In another example using Gemini surfactants, the observed rate constant increased with increasing micelle size, as well as the sphere-to-rod transition. This effect was attributed to changes in the ionization of the micelle, resulting in changes in the interfacial polarity [34].

### 2.4. Effect of Surfactant on Reagent Solubility

In addition to affecting reaction kinetics, surfactants can also increase the catalyst and substrate solubility [19] and solubilize hydrophobic reactants and bases. For example, micelles have been used to facilitate the oxidation of benzylic alcohols in water. The oxidation of 1-(4-methoxyphenyl)methanol resulted in low yields in water (23%) at room temperature. In the presence of surfactant micelles, the yield of the aldehyde generally tripled and approached 97% using PEGylated Guerbet-alcohol-based surfactant micelles [35]. Additionally, to achieve alkali/metal-free catalysis, cyclic guanidine derived bases, such as 1,4-diazobicylco [2,2,2]octane (DABCO), are of interest due to their high basicity. Practically, their use can be limited to difficult separation from the reaction. Surfactant (e.g., SDS) micelles facilitated recovery and reuse of the organic base following a Knoevenagel condensation reaction of salicylaldehyde to 3-acetyl coumarin as a model reaction. The micelles facilitated the reaction by solubilizing the hydrophobic reactants. The water-soluble base interacted at the interface, resulting in enhanced conversion. To reuse the surfactant following the reaction, the reaction mixture was crystallized, the micelles were separated by filtration, and the surfactant and base were reused by adding additional reactants [36]. 

Furthermore, surfactant micelles have been used as alternatives to organic solvents. For example, spirocyclization of keto-ynamide, a reaction typically performed in toluene or THF with metal-based catalysts, was performed using CTAB micelles (ca. 3.6 nm radius) dispersed in water. After 48 h at 30 °C, 100% conversion of the substrate was observed. The desired reaction mixture contained the spiro ester and spiro acid; the desired E isomer of the spiro ester was recovered with a 75% yield [37]. 

To achieve solvent-free processing conditions, lysine has been used as a catalyst for condensation of aldehydes. Treatment of long-chain aldehydes (longer than C6) with 10 mol% lysine for 2 h at room temperature resulted in the desired condensation with a yield of more than 60%. Higher yields were achieved with more hydrophobic substrates. For example, a 74% yield was obtained with nonanal. In this case, the reaction occurred in micelles formed by the reaction intermediate and condensation product [38]. 

Micelles can also enable reactions of hydrophobic substrates in water. For example, photocatalysts, such as iron porphyrin complexes, have been encapsulated in micelles using *N*,*N*-dimethyltetradecylamine *N*-oxide (DTAO) surfactant. Iron (III) meso-tetrakis(2,6-Di-chlorophenyl) porphyrin chloride [Fe(III)(TDCPP)Cl] was encapsulated, and under anaerobic conditions, the iron porphyrin complexes were photochemically reduced to bis(pyridine) hemochrome [Fe(II)TDCPP(py)_2_]. When the photoreduction was carried out in the presence of cyclohexane or cyclooctene, photo-oxygenation of the cycloalkane was achieved. Photoreduction of carbon tetrachloride was also performed with ethanol with high conversion (75%) and turnover (>1500) [39]. Similarly, a ruthenium-based complex, tris(2,2′-bipyridine) ruthenium (II) (RuBBy), a visible-light photoredox catalyst, has been used in SDS micelles. The resulting micelles were used for pinacol couplings of 4-chlorobenzaldehye or benzophenone to form benzylic alcohols [40]. Iridium-based catalysts were investigated to produce amides from *N*-methyl-*N*-alkyl aromatic amines and various organic compounds in water. A model reaction of *N*,*N*-dimethylaniline and toluenesulfonylmethyl isocyanide was performed at room temperature using blue light (16 W) for 48 h. The presence of the micelles resulted in higher yields than using water as the solvent [41]. To enable metal-free photocatalysts, a visible-light catalyst (CN620) for oxidative cleavage of the *C*-*C* bond of vicinal diols in micellar media has been reported. The model substrate was (*R*,*R*)-hydrobenzoin. When water was used as the solvent, a trace amount of the desired product was observed. Introducing surfactant (CTAB) increased conversion. To reuse the catalyst, the final reaction product was obtained by extraction with ethyl acetate. The resulting aqueous media (containing the catalyst) could be reused for at least 10 runs [42]. In another example, peroxidation of methyl linoleate in SDS was performed using mono-azoaromatic photo initiators (e.g., pyridine, quinolone, and purine). The result of the photo-initiated peroxidation was four geometric isomers (9- and 13-positional hydroperoxides) typical of free-radical chain peroxidation. The use of a photosensitizer, such as Methylene Blue or Rose Bengal (expected to result in singlet oxygen), resulted in six hydroperoxides. The use of SDS increased the ratio of 13 to 9, i.e., increased the selectivity of oxidation at the 13 position. This result was attributed to the environment of the micelle structure [40].

### 2.5. Surfactants Incorporating Catalysts

In some systems, surfactants have dual roles as catalysts. For example, 4-dodecylbenzenesulfonic acid (DBSA) has been used as a surfactant and catalyst for synthesis of alkyl glycosides in micelles. Specifically, DBSA micelles (3.9 nm diameter) were effective for the synthesis of chloroalkyl glycosides from glucose and 2-chloroethanol. Chloroalkyl glycoside was obtained with a high yield (90%) and purity (99%), with 94% conversion of the glucose. Hydro-alkyl glucosides, intermediates in the synthesis of bio-based polyols for polyurethanes, were also achieved using DBSA micelles. Ethylene glycol was reacted with glucose, and 2-hydroxyethyl glycoside was the main product. High conversion of glucose (>99%) was observed. The product was recovered with 93% yield and 99% purity [43]. Glucose-derived surfactant (*N*-alkanoyl-*N*-methyl-1-glycamine polyol) has also been used a catalyst for esterification of carboxylic acid and alcohols in aqueous micelles. Without the surfactant micelles, no reaction between benzoic acid and methanol was observed. In the presence of 0.1 mmol surfactant, 65% yield was achieved. The yield of product could be improved by increasing the temperature and decreasing the pH of the reaction [27,44]. Similarly, *p*-dodecylbenzenesulfonic acid acted as a surfactant and catalyst for Biginelli reactions (i.e., synthesis of 3,4-dihydropyrimidin-2-(1*H*)-one derivatives from an aldehyde, β-ketoester, and urea [45]).

Surfactants incorporating catalysts have also been developed. Commonly, the surfactant has contained ligands to complex a metal catalyst (metallosufactant) so that the chemical reaction can be localized to the micelle interface [46]. Metallosurfactants have also been used as precursors to metal nanoparticles, and the resulting nanoparticle-loaded micelles have been used for catalysis (recently reviewed elsewhere [47]). For example, metallosurfactants complexed with palladium and nickel (bisdodecylamine palladium(II) chloride and bisdodecylamine nickel(II) chloride) were micellized (above their CMC) and reduced to Pd-Ni alloy nanoparticles (5 nm) coated in surfactant using sodium borohydride. The resulting surfactant-coated particles were used for Mizoroki-Heck coupling between styrene and iodobenzene using K_2_CO_3_ as a base and water or water-ethanol as the solvent. The reaction proceeded in water–ethanol with microwave heating. Notably, the surfactant coating affected the performance of the coupling reaction. The surfactant was removed by calcination (confirmed by IR), and the yield decreased to 60%, compared to an 86% yield with the surfactant coating. This result suggested that the alkyl chains of the surfactant facilitated transport of hydrophobic reactants from the hydrophilic bulk to the catalytic metal nanoparticles [48].

## 3. Surface-Active Ionic Liquids (SAILs)

In addition to traditional surfactants, other amphiphilic systems can produce micelles. One emerging class of amphiphiles for performing reactions is amphiphilic ionic liquids. Ionic liquids (low-melting-point salts) are considered promising solvents for a wide variety of applications (e.g., biomass processing and lipid extraction from microalgae) [49]. The chemical structure of ionic liquids and their resulting physicochemical features can be tuned by varying their composition (cation and anion) [50]. Substituent groups with long hydrophobic chains (typically longer than eight carbons) and hydrophilic head groups have been termed surface-active ionic liquids (SAILs) [50]. Such systems are amphiphilic with surfactant-like properties. Specifically, they have been observed to form amphiphilic nanostructures, including micelles that can be leveraged for chemical reactions [49]. For example, amphiphilic guanidinium ionic liquids have been used for Suzuki coupling using Pd-based catalysts. Amphiphilic imidazolium ionic liquids have been used for reductive degradation of Rhodamine B, Diels-Alder reaction of 1,3-cyclohexadiene and *N*-benzylmaleimide, Heck coupling of ethyl acrylate and iodobenzene (Pd-catalyzed), and aerobic oxidation of 1-octanol to octanoic acid in water using a Pd-based catalyst. In the case of aerobic oxidation of 1-octanol, the ionic liquid micellar reaction media outperformed a traditional surfactant, CTAB. Ionic liquid can be designed for catalytic purposes. For example, a catalytically active tungstate dianion (WO42-) was used as a counterion for an amphiphilic imidazolium ionic liquid. The resulting micelles were used for epoxidation of olefins in aqueous media [51]. Palladium nanoparticles were stabilized in ionic liquid micelles (PEG-functionalized dicationic ionic liquid (C1_2_Im-PEG IL)). The resulting nanoparticle-loaded micelles facilitated hydrogenation of aliphatics, aromatics, and nitroaromatics to the corresponding alkanes and anilines [52]. 

**Figure 2:**
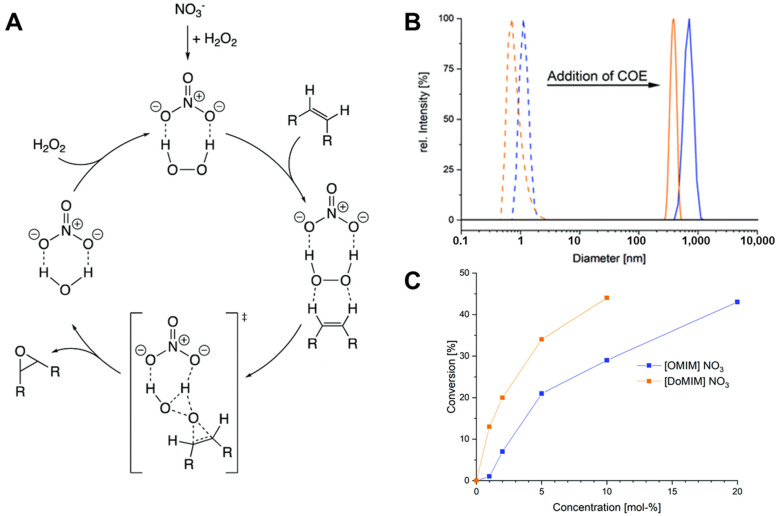
Surface-active imidazolium-based ionic liquids (IL) are known to form micelles in water and are capable of solubilizing cyclooctene to carry out metal-free epoxidation in water catalyzed by H_2_O_2_. (**A**) Proposed mechanism of the activation of H_2_O_2_ and olefin epoxidation by the nitrate anion. (**B**) Micelle size in 50 wt% H_2_O_2_ at 80 °C measured by dynamic light scattering. Blue: 357 mM [OMIM][NO_3_]. Orange: 357 mM [DoMIM][NO_3_]. Dashed line: IL solutions with ‘empty’ micelles; solid line: micellar solution after saturation with cyclooctene as model substrate. (**C**) Conversion of cyclooctene in the epoxidation with [OMIM][NO_3_] (blue) and [DoMIM][NO_3_] (orange). Product separation was achieved by decanting the supernatant phase. Adapted with permission from [53].

To facilitate metal-free transformations of olefins to epoxides, imidazolium nitrate micelles have been used with aqueous hydrogen peroxide to perform epoxidation reactions. Following dynamic light scattering, the micelles of [OMIM][NO_3_] were approximately 1 nm. The addition of cyclooctene caused the micelles to swell to more than 500 nm (Figure 2). Thus, the micelles solubilized approximately two molecules per [OMIM][NO_3_] pair. Using a reaction temperature of 80 °C, the conversion of substrate after 24 h was 27%, with a selectivity for the epoxide of 74%. The only byproduct was cyclooctanediol. Higher conversions were achieved by increasing the temperature. Product separation was achieved by decanting the supernatant. The ionic liquid in the residual aqueous phase could be reused by adding additional oxidant and substrate. Notably, when the reaction was performed under the same conditions with salts that do not form micelles (i.e., NaNO_3_), the conversion was 6%. This comparison demonstrated that the performance of the catalyzed reaction was enhanced by the presence of the micelles [53]. 

## 4. Designer Surfactants

**Figure 3:**
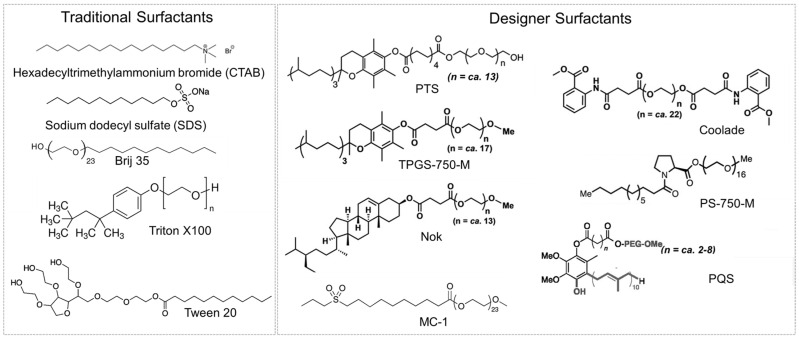
Structures of traditional surfactants and designer surfactants commonly used for organic reactions in water.

Another class of amphiphiles used to perform reactions in water is “designer surfactants”. “Designer” surfactants have been defined as new amphiphilic molecules designed specifically for catalytic applications in water [27,54]. Lipshutz and colleagues developed a series of “designer surfactants” (Figure 3). The properties of “designer” surfactants are comparable to those of conventional surfactants. For example, Triton X100 is a nonionic conventional surfactant with a hydrophilic lipophilic balance (HLB) of 16.7 and a critical micelle concentration of 0.23 mmol [55]. “Designer” surfactant TPGS-750 is nonionic, with an HLB of 13 [56] and a critical micelle concentration (CMC) on the order of 0.1 mmol [57]. For reactions, one important difference between “designer” surfactants and conventional surfactants is the resulting micelle size. Compared to conventional surfactants, “designer” surfactants typically form larger micelles. For example, Triton X100 forms micelles on the order of ~7.5 nm [58], whereas TPGS-750-M forms micelles on the order of ~50 nm in diameter [56]. Micelles 50 to 60 nm in diameter can improve apparent reaction performance compared to smaller micelles (~10 nm) [9]. A comparison of the properties and micelle sizes of conventional and designer surfactants is provided in Table 2.

Overall, nonionic surfactants have enabled a wide scope of reactions in terms of the type of transformation and substrate variation. Such nonionic surfactants include PEGylated amphiphiles, such as TPGS-750-M and PTS, which yield spherical micelles, as well as Nok, which yields rod-like micelles (Figure 1). Micelles with dimensions on the order of 50–60 nm are considered ideal for accommodating exchange of reactants, products, etc., with a sufficient hydrophobic microenvironment to facilitate the reaction [62].

Micelles of designer surfactants in water can facilitate reactions typically performed in organic solvents (examples of reactions summarized in Table 3) [63]. For example, olefin metathesis is a widely used transformation for the formation of carbon–carbon double bonds. Typically, such reactions are carried out under anhydrous conditions in chlorinated solvents. Designer surfactants, such as polyoxyethanyl α-tocopheryl sebacate (PTS), have enabled such transformations using water as the solvent and ruthenium-based catalysts at room temperature. Five-to-seven-membered rings and tri-substituted alkenes were produced. Following the reaction, the product and catalyst were extracted with ether. The PTS remained in the aqueous phase. In subsequent cycles, additional substrate and catalyst were added [64]. 

Similarly, d-α-tocopheryl polyethylene glycol succinate (TPGS-750-M) has been used as an alternative to organic solvents for reactions. In particular, TPGS-750-M has been shown to function as a replacement for polar, aprotic solvents. TPGS-750-M micelles have enabled many types of reactions, including, for example, transition metal cross couplings, zinc-mediated reductions, Suzuki-Miyaura couplings of MIDA boronates, Stille couplings of alkenyl and aryl halides, and aerobic oxidations of alcohols, which have been previously reviewed (brief overview in Table 3) [6,16,65,66,67]. Metal nanoparticles can be combined with TPGS-750-M micelles. For example, using copper nanoparticles, click reactions have been performed in water at room temperature in micelles. Using Pd catalysts, Suzuki-Miyaura coupling, Lindlar reductions, and nitro-group reductions have been achieved. TPGS-750-M increased the reactivity of palladium nanoparticles on activated carbon, as indicated by the fivefold increase in yield in the Suzuki-Miyaura cross-coupling reaction between 4-bromoanisole and phenylboronic acid [68]. Palladium nanoparticles and TPGS-750-M have also been used for reduction of alkynes. A *Z*-olefin product was achieved with excellent yield (95:5 *Z*-to-*E* ratio). Monosubstituted alkenes were achieved from terminal alkynes. The products were isolated by extraction, and the remaining aqueous phase containing the micelles and catalysts could be reused [69]. To minimize the use of precious metals, iron-based particles have been used with micellar media. For example, iron particles have been produced with small amounts of copper (1000 ppm). The resulting particles could be dispersed in water using TPGS-750-M and used for alkyne-azide click chemistry to produce 1,4-distributed triazoles. “In-flask” extraction with ethyl acetate was used to isolate the product. The E factor for solvents used in this process was approximately four. For reuse, the catalyst and micelles, vitamin C, and additional reactants were added. The catalyst could be reused at least three times, maintaining 95% isolated yield of the desired product [70]. Carbonyl iron powder has been used with TPGS-750-M for nitro-group reductions in water, whereas the same reactions cannot be achieved in organic solvents (DMF, THF, or MeOH) [9]. 

The performance of Nok micelles is comparable to that of systems that form spherical micelles for many classes of reactions, including olefin metathesis, Suzuki-Miyaura coupling, Heck coupling, aminations, etc. [65]. A lipophilic ligand to complex with Pd (HandaPhos) was developed and used with Nok surfactant. Due to its lipophilic nature, the ligand was incorporated into the core of the micellar structure, and the effective concentration during reaction was enhanced. Thus, only ppm levels of catalyst were needed for Suzuki-Miyaura and Sonogashira couplings. Gold-catalyzed reactions (cycloisomerization of allenes) were also achieved [9]. Reactions performed with Nok are summarized in Table 3.

A new surfactant to accommodate polar substrates (MC-1) was developed for peptide synthesis. The lipophilic tail of MC-1 contained sulfone groups, and the resulting micelle core accommodated polar amino acids, nitroalkanes, and hydrazones. This designer surfactant provided a promising alternative to dipolar aprotic organic solvents (e.g., DMF and NMP). Micellar reaction media using MC-1 have also increased the apparent reaction rate of enzymes (e.g., keto reductase ADH101 using (*E*)-4-phenyl-3-buten-2-one). The micelles were thought to act as a reservoir for the educts and products, regulating their concentration by enabling dynamic exchange with the enzymatic pocket, increasing accessibility of the substrate. The result was improved conversion and isolated yields with increasing surfactant concentrations [16,61]. 

Surfactants that do not foam under reaction conditions, e.g., Coolade, have also been investigated by decreasing the length of the hydrocarbon chain. This feature is advantageous for reactions that have a gaseous byproduct, e.g., nitro-group reductions using sodium borohydride [15,54]. 

**Figure 4:**
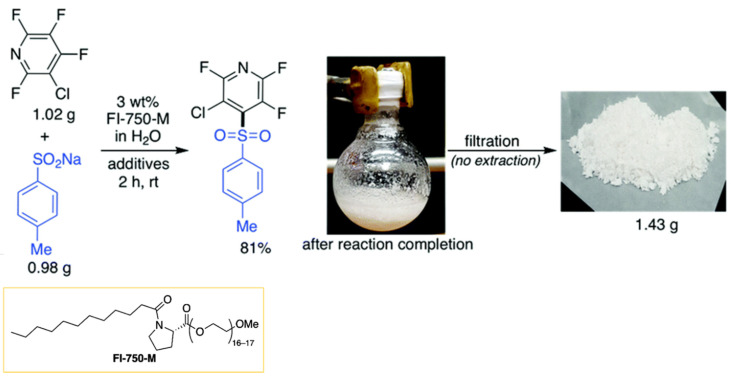
Gram-scale reaction of polyfluoroarene and sodium *p*-toluenesulfinate salt using FI-750-M micelles. The resulting product was recovered as a solid by filtration, so no organic solvent was required for extraction and purification. Adapted from [71] with permission.

Handa and colleagues engineered FI-750-M, a proline-based designer surfactant (Figure 4) that mimics polar organic solvents, such as DMF and dioxane. In an aqueous environment, FI-750-M formed micelles with an inner lipophilic region, a proline linker, and a mPEG region at the interface with the aqueous solvent, with binding sites for polyfluoroarenes and sulfonate salts. Such binding was leveraged for sulfonylation of polyfluoroarenes under ambient conditions using water as the bulk solvent. As a model reaction, pentafluorobenzonitrile was reacted with sodium *p*-toluenesulfinate salt at room temperature (Table 3). The yield of the product in the presence of FI-750-M micelles was higher than that of other surfactants (TPGS-750-M, SDS, Tween 20, and Pluronic F-127). An additive (NaCl) was used to increase the product yield. This effect was attributed to enhanced exchange between dynamic micelles. The reaction between polyfluoroarenes and *p*-toluenesulfinate salt could be performed on a gram scale. The product precipitated from the aqueous dispersion and was recovered by filtration and washing (Figure 4). The resulting product could be polymerized, and the resulting polymers have potential applications as membranes for gas separation. The aqueous phase containing the micelles was reused in subsequent reactions [71].

For photocatalysis, the designer surfactant PQS photocatalyst has been used as a platform. PQS contains a lipophilic portion (50-carbon side chain), a hydrophilic portion (PEGylated succinic acid conjugated to CoQ10), and a free –OH group to link photocatalysts (e.g., fac-Ir(ppy)3). Upon self-assembly, the photocatalyst is confined to the hydrophobic core of the micelle. The resulting micelles have been used for photoreactions between alkenes and sulfonyl chloride (e.g., difunctionalization of α-methylstyrene to β-hydroxysulfone). Yields of 90% were achieved using 1 mol% catalyst, blue LED (5 W), under argon after 18 h. A variety of benzenesulfonyl chlorides with electron-donating, -neutral, and -withdrawing groups at the para position were also reactive. Heteroaryl sulfonyl chlorides also reacted. Reaction of alkyl sulfonyl chlorides resulted in modest yields, even when excess substrate (3 eq.) was used. Sulfonylation of enol acetates was also achieved. To recycle the photocatalyst, extraction was performed with ether. The resulting aqueous phase containing the catalyst could be reused as many as four times before adding additional catalyst. Notably, conjugation of the catalyst to the micelle facilitated catalyst reuse [41]. Alternatively, gold(I) complexes were conjugated to the PQS. Full dehydratative cyclization was achieved in 4 h using acetylenic diol was used as a model substrate. The hydrophobic core of the micelles was thought to favor product formation. The addition of SDS as a cosurfactant accelerated the apparent rate. Full conversion was achieved within 5 min. The product was recovered by in-flask extraction. The catalyst could be reused twice following the extraction. Notably, SDS was thought to limit the stability of the catalyst and complicated phase separation during product extraction. Without SDS, the catalyst could be reused seven times. Although some loss in reactivity was observed as the time required for full conversion increased from 5 to 20 h [72]. Similarly, Lipshutz and colleagues used the PQS platform with a ruthenium-based catalyst (a Hoveyda-Grubbs catalyst). In water, the resulting surfactant self-assembled into micelles (44 nm). The resulting micelle-containing catalysts efficiently performed ring closing metathesis of five-to-seven-membered rings in water or ocean water. Conversions of as much as 99% at room temperature were achieved. The product could be separated from the catalyst by extraction with ether. The resulting aqueous fraction containing the catalyst could be reused [73]. A brief summary of the reactions performed using PQS as a platform is included in Table 3.

## 5. Block Copolymer Micelles

### 5.1. Polymer Micelle Background

In addition to designer surfactants, another class of customizable amphiphiles is block copolymers that self-assemble into micelles. Such self-assembled polymeric micelles dispersed in water have also been used as reaction media for organic chemistry. Many classes of polymer nanostructures (e.g., dendrimers, polymersomes, nanogels, etc.) have been studied as nanoreactors for chemical reactions and are described elsewhere [5,74,75,76]. Polymer micelles are a relatively simple, common, and well-defined structure that can be achieved via self-assembly of amphiphilic block copolymers. Thus, here, we focus on amphiphilic polymer micelles (spherical or rod-shaped) analogous to small-molecule surfactant systems.

To achieve polymer micelles in water, the amphiphilic copolymer is typically dissolved in an appropriate solvent for both blocks; then, water, a non-solvent for the hydrophobic block, is added to cause aggregation of the hydrophobic blocks driven by a decrease in free energy. The resulting structure is a well-defined spherical micelle with a hydrophobic core surrounded by a hydrophilic shell dispersed in water. The micelles can be kinetically trapped by replacement of the appropriate solvent with non-solvent through dialysis. Kinetic trapping prevents dynamic exchange of the polymer chains. The properties (size, shape, and material) of polymeric micelles can be readily tuned. For example, diameters in the range of 10 to 100 nm can be obtained by varying the block lengths. Such polymeric micelles are promising systems for organic reactions; ca. 50 nm hydrophobic pockets have provided a unique reaction environment with improved selectivity compared to a small-molecule micelle. This effect was attributed to the stability of the confined hydrophobic pocket [7,77].

Similar to small-molecule surfactant micelles, a hydrophobic polymer micelle core can enhance the solubility of organic compounds in water. The high local concentration in the core, for example, can enhance reaction rates. Additionally, the hydrophobic polymer core limits water, which increases the stability of substrates sensitive to hydrolysis. Because the polymeric nanoreactors are stable (compared to small-molecule micelles), the catalyst can be recovered following the reaction by precipitation or ultrafiltration methods. Through selection of the chemical moieties on the polymer, the selectivity can be modified based on non-covalent core-substrate interactions, e.g., hydrogen bonding [78]. Tools in polymer chemistry provide a platform to impart block copolymer micelles with unique properties, e.g., selective crosslinking to impart mechanical stability for reuse, stimuli-responsive components (e.g., pH and temperature), and the ability to incorporate a range of catalysts, such as organocatalysts, metal nanoparticles, and metal complexes [8,79].

### 5.2. Polymer Micelles Functionalized with Organocatalysts

To utilize organocatalysts with polymer micelles, the catalyst can be directly incorporated into the polymer backbone via polymer synthesis. For example, 4-dimethylaminopyridine (DMAP), a nucleophilic catalyst used for a variety of reactions, such as esterification with anhydrides, has been incorporated into amphiphilic block copolymers micelles. DMAP was copolymerized with styrene using reversible addition-fragmentation chain-transfer (RAFT) polymerization to create a hydrophobic block containing catalyst, and the block was chain-extended with a water-soluble poly(N-isopropylacrylamide) (NIPAM) block. The resulting diblock copolymer self-assembled into kinetically trapped micelles in water (approximately 24 nm in diameter). Esterification of 1-phenylpropanol with a 1:1 mixture of acetic anhydride and butyric anhydride was examined. In the presence of the micelles, more than 80% conversion was achieved. The reactivity with butyric anhydride was higher than that with acetic anhydride (the opposite of what was observed with unsupported DMAP alone). This result was attributed to the hydrophobicity of the micellar core. Leveraging this microenvironment, linalool was reacted with methanol. Acylated linalool was observed within 1 h in the presence of micelles but was not achieved if DMAP was used without micelles. In this case, the hydrophobicity of the micelle core facilitated the reaction of otherwise non-reactive alcohols by bringing the reactant in closer proximity to the catalyst [80].

Another organocatalyst that has been incorporated into micelles is proline. Amphiphilic polymers containing proline have been synthesized by copolymerizing styrene with a functionalized monomer containing proline moieties. The resulting polymers self-assemble in water and can be used for asymmetric aldol reaction between cyclohexanone and *p*-nitrobenzaldehyde. Compared to unsupported proline, lower amounts of catalysts can be used. The product was recovered by extraction with ethyl acetate and purified by column chromatography. The polymer aggregates precipitated from the aqueous phase following the reaction (and quenching with addition of LiBr) and could be dried and reused. Recovery of the catalyst was approximately 90% in each cycle. Higher-molecular-weight polymer catalysts led to more efficient recoveries. Thus, whereas the polymer molecular weight did not affect the catalytic performance, it did influence the reusability [81]. Alternatively, proline moieties were added to amphiphilic polymers using post-polymerization modification. Specifically, the azlactone-containing PEG-b-(PNIPAAm-co-VDMA) was covalently attached to amine-functionalized, N-boc-protected *trans*-4-hydroxy-L-proline. Upon micelle formation in water (ca. 50 nm) at elevated temperatures (>32–36 °C), the immobilized catalyst was located at the hydrophobic/hydrophilic interface. The resulting block copolymer micelles were used for asymmetric aldol reaction between cyclohexanone and *p*-nitrobenzaldehyde with quantitative aldehyde conversion. After the reaction, the substrates and products were extracted with diethyl ether. The block copolymer micelles remained in the aqueous phase and could be recovered by freeze drying. Examining reusability for five cycles, the loss of polymer after each catalyst cycle was 4–8 wt%. Substrate conversion was not affected with reuse. However, a decrease in enantioselectivity and product yield was observed with multiple cycles [82]. 

Other catalytic moieties, such as sulfonic acid groups, have also been incorporated into block copolymers. Amphiphilic polymers (copolymers of hydrophilic poly(*N*,*N*-dimethylacrylamide) and hydrophobic poly(n-butyl acrylate)) with hydrophobic blocks functionalized with sulfonic acid have also been developed. The resulting polymer-formed micelles (ca. 20 nm in diameter) in aqueous media were assumed to contain sulfonic acid moieties in their cores, which can be used for Brønsted acid catalysis. For example, the micelles facilitated the conversion of DNA-conjugated aldehydes to substituted tetrahydroquinolines by Povarov reaction (of DNA-conjugated aldehydes, aniline, and isocyanides with aniline and olefin) without depurination. Aminoimidazopyridines (e.g., DNA-tagged 3-aminoimidazo[1,2-a]pyridines) were also synthesized by Groebke-Blackburn-Bienayme reaction and Boc deprotection. The products were recovered by extraction with ethyl acetate. This example demonstrates that block copolymer micellar reaction media can be used for synthesis of DNA-tagged molecules and may be a useful tool for DNA-templated chemistry for screening library synthesis [83].

### 5.3. Stimuli-Responsive Polymer Micelles

To achieve systems with tunable or switchable catalytic activities and solubilities, stimuli-responsive micelles have been developed. Temperature-responsive polymers based on NIPAM have proven to be a versatile platform for such responsive micelles. For example, proline-containing monomers were incorporated into an amphiphilic block copolymer with poly(dimethylacrylamide) hydrophilic block with a hydrophobic copolymer of NIPAM and a proline-containing monomer. The resulting polymers were thermoresponsive with temperature-dependent solubility. The polymer was soluble in water at low temperatures. Above a phase-transition temperature, the hydrophobic block became insoluble, and micelles with a hydrophobic NIPAM core formed. Solubilization of the polymer to release and recover the product was achieved by decreasing the temperature. Following the asymmetric aldol reaction between cyclohexanone and *p*-nitrobenzaldehyde performed at 50 °C, the reaction mixture was cooled, and the precipitated product was collected by centrifugation. The remaining aqueous phase (containing the polymer) was reused. Upon reuse, a decrease in conversion was observed (85 to 68% after five cycles). No change in stereoselectivity was observed for reactions performed at 50 °C [84]. In another example, block copolymers with a PNIPAM-based block (hydrophilic) with a proline-containing hydrophobic block were synthesized through cationic ring-opening polymerization. The polymers self-assembled into micelles in water. The hydrophobic catalyst was surrounded by a hydrophilic shell. The confined catalyst was used for an aldol reaction between 4-nitrobenzaldehyde and cyclohexanone. At 30 °C, the yield was 99% with 94% ee (enantiomeric excess). The effect of nanoreactor size was investigated using a series of block copolymers. In general, smaller micelles improved stereoselectivity, which was attributed to optimized water transport to the hydrophobic core. To reuse the micelles, the products and remaining reactants were removed by extraction with chloroform, and the chloroform was removed under vacuum. The yield decreased with every reuse, and selectivity decreased after the third reuse. The apparent decrease in catalytic efficiency and selectivity was attributed to the loss of micelles and disruption of the micelle structure during extraction [85]. The performance of micellized block copolymers was compared to that of random copolymers that to did not form micelles. Whereas the yield was comparable, the selectivity was higher when using the system that formed micelles (96% ee compared to 76% ee). The high selectivity of the micelles was attributed to the hydrophobic microenvironment of the core [86]. A similar approach was used to incorporate imidazole moieties into temperature-responsive micelles. Poly(*N*-isopropylacrylamide)-b-poly(*N*-vinylimidazole) (PNIPAM-b-PVim) was prepared by RAFT polymerization. The copolymers were soluble in aqueous solvent mixtures at room temperature. Above the phase transition, the PNIPAM block becomes hydrophobic, and the block copolymer forms spherical micelles with PNIPAM cores and PVim shells. The size of the micelle depends on the molecular weight of the polymer. The resulting polymer was used for hydrolysis of 4-nitrophenyl acetate to *p*-nitrophenol. Examining the reaction rate as a function of temperature, enhancement of the catalytic activity was observed above the phase-transition temperature. This rate enhancement was attributed to micellization of the polymer, resulting in increased local concentrations of the substrate [87].

Similarly, proline has been incorporated into the hydrophobic backbone of pH-responsive, amphiphilic, PEG-based block copolymers by RAFT polymerization using diethylaminoethyl methacrylate (DEA) as a pH-responsive monomer. The resulting amphiphilic block copolymers self-assembled into micelles in water. The micelle size was proportional to pH (increasing from 18 to 220 nm between pH 4 and 9). The increase in size was attributed to deprotonation of the polymer, resulting in increased hydrophobicity of the DEA segments. Using a model reaction of cyclohexanone and *p*-nitrobenzaldehyde, conversion of *p*-nitrobenzaldehyde (93%) and yield was highest (75%) at pH 7. This effect was attributed to the hydrophobic pocket compared to lower pH values and mass-transfer limitations at higher pH values. Following the reaction, CO_2_ was introduced to disassemble the micelles, and the product was extracted using ether and further purified by column chromatography [88]. 

Light-responsive micelles have been achieved based on an amphiphilic poly(2-oxazoline) diblock copolymers functionalized with spiropyran. Cationic ring-opening polymerization was used to synthesize poly(2-oxaline) diblock copolymers with alkyne side chains on the hydrophobic block. Using copper-catalyzed azide-alkyne click chemistry, azide-functionalized spiropyran was attached to the polymer. Upon self-assembly in water, the polymer formed spherical micelles that transition to vesicles when exposed to UV light. The structural transition was attributed to an increase in hydrophilicity of the polymer chain when the spiropyran isomerizes to merocyanine The transition was reversible, and micelles reformed upon radiation with visible light. TREN (tris(2-aminoethyl) amine, a catalyst, was incorporated so that it resided at the hydrophilic/hydrophobic interface. The effect of the structural switch on catalytic performance was examined. For example, the micelles were used for Knoevenagel condensation between nitrobenzaldehyde and malononitrile in chloroform and in the presence of benzyl alcohol. Condensation proceeded in the dark, with 70% conversion. Upon exposure to UV, apparent reaction kinetics increased. This effect was attributed to enhanced transport of the substrate after the switch from micelles to vesicles. Polymers could also be designed to precipitate out of solution upon exposure to UV light, stopping the reaction [89].

### 5.4. Polymer Micelles Functionalized with Nanoparticle-Based Catalysts

As alternatives to organocatalysts, polymer micelles incorporating metal-based catalysts (e.g., metal nanoparticles) have been used for hydrogenation, oxidation, reduction, and Heck reactions. In some cases, the polymer can be crosslinked to enhance the stability of the micelles under various process conditions (temperatures and solvent concentrations). Either the core or shell component can be covalently crosslinked. Such crosslinking strategies may enable reactions that are incompatible or currently unachievable in water to take place within the hydrophobic core of the polymeric micelles dispersed in the aqueous solution [7,77].

For example, gold nanoparticles have been incorporated into polymer micelle structures for catalytic applications using polyethylene oxide-b-polyacrylic acid (PEO-b-PAA) block copolymer micelles. The block copolymer was dissolved in water with HAuCl_4_. The gold precursor acts preferentially with the carboxylate of the PAA block and aggregates into micelles. Hydrazine was then added to reduce the gold. The micelles act as a template for the growth of gold nanoparticles within the core of the micelle. Thus, the PEO block sterically stabilizes the nanoparticles. The resulting dispersion was centrifuged and dialyzed. The resulting gold nanoparticles had an average diameter of 10.0 ± 4.0 nm after centrifuging. The resulting micelles containing gold nanoparticles facilitated the reduction of 4-nitrophenol with sodium borohydride, with a turnover frequency of 800 h^−1^. Similarly sized citrate-stabilized gold nanoparticles had a turnover frequency of 570 h^−1^. The authors attributed the enhanced catalytic performance to the surrounding block copolymer environment. Reduction of 2- and 3-nitrophenols was also possible [90]. Similarly, gold clusters were prepared in PEG functionalized with hexene groups self-assembled into micelles. The micelle core was crosslinked with thiol-ene “click” chemistry. Depending on the size of the PEG chain, the micelle diameter could be tuned between 12 and 90 nm (measured after crosslinking). Upon addition of a gold precursor, gold nanoclusters (<1.0 nm) formed throughout the core and shell of the micelle, as indicated by the fluorescent properties at 365 nm. The resulting gold-containing micelles were used for aerobic oxidation of benzoin in water. Complete conversion was observed at 50 °C after 14 h. Aryl α-hydroxy ketone derivatives could also be oxidized. To reuse the micelles, the aqueous phase was extracted with ether. The catalyst could be reused as many as 48 times, with negligible changes in the turnover frequency. No formation black precipitate was observed. Thus, the PEG layers of the micelle were thought to prevent aggregation of the gold clusters and promote reusability of the catalyst [91].

With the aim of modular material selection (polymer components and metal nanoparticle), flash nanoprecipitation has been used as a platform for self-assembly of an amphiphilic block copolymers, metal nanoparticles, and hydrophobic coprecipitants. The block copolymer micellized and directed self-assembly of a filled micelle. The hydrophobic coprecipitant and metal nanoparticle were incorporated into the core of the core-shell micelle structure. Using dodecanethiol-capped gold, Harrison et al. demonstrated that the size of the nanoreactor and metal nanoparticle loading could be tuned independently. The kinetics of the encapsulated gold nanoparticles were studied using reduction of *p*-nitrophenol in the presence of sodium borohydride as a model reaction. The induction time was longer than that of citrate-capped gold nanoparticles, and the rate constant was comparable to that of ligand-free gold nanoparticles. Thus, diffusion and partitioning of sodium borohydride affected the induction time. Given sufficient equilibration time, the intrinsic kinetics of the catalysts were not affected by incorporation into the micelle, and further mass transfer affects where not observed [92]. The selection of the coprecipitant was modular and included polystyrene or castor oil. The reaction rate normalized to the surface area of gold nanoparticles was more than eightfold higher in castor oil than in polystyrene. This rate acceleration was attributed to enhanced solubility of the reactants in the hydrophobic microenvironment rather than differences in mass transfer or intrinsic kinetics. The nanoreactors could be reused for multiple reactions; full conversion of the 4-nitrophenol was achieved within 3 min for at least 10 sequential reactions [93]. Building on these results, the nanoreactors (with a polystyrene coprecipitant) were used for a reaction between benzaldehyde and 4-nitrophenol. The product (solid) phase separated from the nanoreactor dispersion and could be recovered via filtration. A 65% isolated yield of 4-benzylideneaminophenol was achieved [94].

Other types of metal nanoparticles have also been encapsulated within polymer micelles. For example, copper(I) nanoparticles were formulated in polydiacetylene micelles (photocrosslinked) and used for Huisgen [3 + 2] cycloaddition in water. Specifically, oleic-acid-stabilized cuprous oxide (Cu_2_O) nanoparticles (average diameter, 8.6 ± 1.8 nm) were encapsulated in the core of self-assembled PEG-b-polydiacetylene micelles. The resulting micelles (30 nm diameter) were UV-crosslinked by polymerization of the diacetylene units in the nanoparticle core. Benzyl azide and phenylacetylene were reacted in water containing the resulting copper nanoparticle-loaded micelles. The triazole product was obtained with a 99% yield. The micelles were more efficient than the copper (II) sulfate and excess sodium ascorbate typically used for click chemistry. To reuse the micelles, the aqueous phase was reused following extraction. Full conversion was observed after five consecutive cycles [95]. Palladium nanoparticles (ca. 20 nm in diameter) were incorporated in the polysilane core surrounded by a poly(methacrylic acid) shell and used for hydrogenation and Heck reactions. Notably, for hydrogenation, the catalytic performance was dependent on the size of the substrate. Whereas 3-buten-2-ol and 2-methyl-3-buten-2-ol did not react, hydrogenation of 1-hexene was observed under the same conditions, with 82% yield [96,97]. Clusters (0.7 nm, approximately seven atoms) of Pd atoms could also be incorporated into shell-crosslinked micelles using ligand exchange with Pd(PPh_3_)_4_. The resulting micelles could be used for hydrogenation of quinolone to 1,2,3,4,-tetraquioline at atmospheric pressure and temperature. Heck reactions could also be performed at low Pd loadings (0.001 mol%). Low leaching confirmed that the Pd was encapsulated within the hydrophobic core of the micelles [97]. 

### 5.5. Polymer Micelles Functionalized with Metal Complex-Based Catalysts

Polymer micelles have also proven a versatile platform for the use of metal salts and metal complexes as catalysts. For example, poly(2-oxazoline) block copolymers polymerized by cationic ring-opening polymerization of 2-heptyl-2-oxazoline (hydrophobic) and 2-methyl-2-oxazoline (hydrophilic) were used with AuBr_3_ for gold-catalyzed cycloisomerization of allene. The resulting micelles were 10 ± 2 nm in diameter. Complete conversion of allene was observed within 6 h with 92% yield (following filtration, washing with ethanol, and purification by flash column chromatography) [98]. 

Metal complexes are also versatile catalysts that have been used with polymer micelles. Leveraging polymer–metal interactions, metal complexes can be incorporated into polymer micelles using self-assembly. For example, sulfur-carbon-sulfur pincer Pd catalysts have been self-assembled with an amphiphilic poly(acrylic acid) (PAA). The resulting micelles were used for Suzuki-Miyaura coupling. Using 2% of the micelle-based pincer catalyst, the apparent reaction rate between vinyl epoxide and phenylboronic acid was 100 times higher than that achieved using an unsupported Pd-complex in organic solvent. The accelerated reaction rate was attributed to the micelle size and hydrophobic pocket. The product was isolated by extraction [5,99]. In another example, Pd complexes were used in the presence of PNIPAM-based block copolymers, which formed micelles above a lower critical solution temperature. The polymer was micellized in water in the presence of PdCl_2_(PPh_3_)_2_ and reactants for Mizoroki-Heck reactions (e.g., iodobenzene and butyl acrylate) by increasing the temperature from room temperature to 70 °C (lower critical solution temperature, 40–50 °C). Following the reaction, the mixture was cooled, and the product was extracted with diethyl ether. The product was further purified by column chromatography. The yield was 99%, which is higher than that achieved using a traditional surfactant, such as SDS (yield 47%) [100]. Copper metal complexes have been incorporated into the hydrophobic core of micelles using an amphiphilic block copolymer with terpyridine in the core. The carboxylic acid functionalities of the intermediate shell were crosslinked via amidation chemistry following self-assembly in water. The resulting copper complex containing micelles was used to catalyze the 1,3-dipolar cycloaddition of azido- and alkynyl-functionalized small molecules [97,101]. 

**Figure 5:**
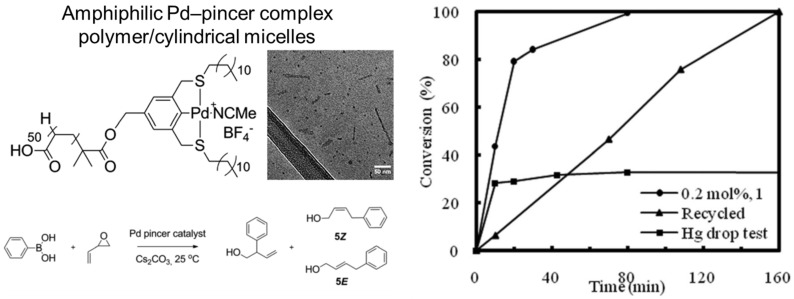
Amphiphilic polymer bearing an SCS pincer palladium complex synthesized by reversible addition fragmentation chain-transfer (RAFT) polymerization. The resulting amphiphile self-assembled into cylindrical (worm-like) micelles in water. The resulting catalyst was used for cross coupling of vinyl epoxide with phenyl boronic acid to afford branched and linear alcohols. The reaction was completed with 0.2 mol% catalyst (100% conversion) after ca. 80 min. Degradation of the catalyst was observed upon recycling of the catalyst and with the addition of Hg(0) (which selectively binds Pd(0)). Adapted from [99] with permission.

Another approach to incorporating metal complexes is to utilize tools in polymer synthesis. Specifically, functional handles for attachment of metal complexes can be incorporated directly into the block copolymer. For example, O’Reilly and colleagues used RAFT polymerization to synthesize a hydrophobic SCS pincer-functionalized block attached to a poly(acrylic acid) (PAA) block. The hydrophobic SCS pincer block facilitated simultaneous complexation to palladium and self-assembly to Pd-loaded micelles. The micelle structure was observed with cryo-TEM (Figure 5). Both spherical and worm-like assemblies were observed. The diameter of both was 5 nm, and the Pd was contained in the core of both types of self-assembled structures. The micelles were used for Suzuki-Miyaura coupling using phenylboronic acid and vinyl epoxide as a model reaction, with water as the bulk solvent. Full conversion was observed in less than 20 min. The reaction was 100 times faster using the polymer micelles than the small-molecule Pd complexes. This increase in apparent rate was attributed to the hydrophobicity of the core of the self-assembled structures, which solubilized the catalyst and hydrophobic substrates. The apparent reaction rate was affected by polymer concentration, and the turnover frequency increased with decreasing polymer concentration. This result indicated that the reaction was limited by the rate of substrate transport into the core. Separation of the reactants and micelles was achieved by extraction with chloroform (as the PAA block was immiscible). However, extraction resulted in a decrease in apparent catalytic activity. This decrease in activity was attributed to 40% of the catalyst being converted from Pd(II) (catalytically active) to Pd(0) (indicated by ICP-OES) following extraction (Figure 5) [99]. In another example, amphiphilic poly(2-oxaloine) block copolymers containing bipyridine pendant groups were achieved by synthesizing 2-oxazoline monomers conjugated to bipyridine. The resulting polymers formed micelles in water (8–21 nm by dynamic light scattering). The resulting micelles were complexed with Cu(I) and used to perform aerobic oxidation of alcohols at room temperature and under ambient pressure in the presence of TEMPO. Conversion of benzyl alcohol to benzaldehyde was 98% after 3 h, with a yield of 94% (turnover frequency of 13.3 h^−1^). The product was isolated by extraction with diethyl ether and purified by column chromatography. The catalyst remained in the aqueous phase and could be reused with additional TEMPO [102].

In another approach to incorporate metal complexes, functional handles on specific blocks of the block copolymer have been leveraged to covalently attach metal complexes following polymer synthesis. For example, a Rh-based Hoveyda-Grubbs catalyst was covalently bound to the hydrophobic block of an amphiphilic block copolymer. Specifically, a poly(2-oxazline)-based amphiphilic block copolymer was synthesized by living cationic polymerization of 2-methyl-2oxazoline, 2-nonyl-2-oxazoline, and ester-substituted 2-oxaxoline monomers. Following a hydrolysis reaction, the hydrophobic block had pendant carboxylic acid groups on the side chain. A 2-isopropoxy-5-hydroxystyrene was attached to the carboxylic acid via a carbodiimide coupling reaction in the presence of DMAP. The second-generation Grubbs catalyst was immobilized at the phenolic group of the attached hydroxystyrene. The resulting catalyst was used for ring-closing metathesis of diethyl diallylmalonate. The product and unreacted starting material were extracted with pentane. Minimal (less than 1 ppm) catalyst was detected in the product by ICP. Using the micelles in water increased the conversion compared to performing the reaction in organic solvents (DMF or DCM). Specifically, a conversion of 90% was achieved using the polymer micelles in water after 1 h at 25 °C using 1 mol% catalyst compared to 48% conversion in DMF under the same conditions. The increase in conversion was attributed to micelle formation. The catalyst could be reused for multiple cycles by adding additional diethyl diallylmalonate. However, the conversion decreased to 9% after five cycles. The decrease was attributed to catalyst deactivation rather than attrition of the catalyst during extraction [103]. Similarly, amphiphilic poly(2-oxaline)s with pendant N-heterocyclic carbene moieties in the hydrophobic block were synthesized and used to complex Pd catalysts. The resulting micelles were used to perform Heck reactions of iodobenzene and styrene in water. Using K_2_CO_3_ as a base, the conversion of iodobenzene after 1.5 h at 90 °C was 97%, and the yield of the *trans*-stilbene was 93%. The turnover frequency was 530 h^−1^. When the reaction temperature was increased to 110 °C, the turnover frequency increased fivefold to 2700 h^−1^. Suzuki reactions, e.g., 4-bromobenzyaldehyde and phenylboronic acid, could also be performed in water using the polymer micelles [104]. Similarly, Co(III) salen complexes have been incorporated into the hydrophobic block of an amphiphilic block copolymer. The salen ligand was immobilized on the carboxylic acid groups of the polymer, and the Co(III) complex was introduced to the ligand onto the polymer chain (one Co atom per polymer chain). In water, the resulting polymer aggregated into micelles with a hydrodynamic size of 10 to 12 nm (radius, 14.3 nm as measured by TEM). The resulting micelles were used for hydrolytic kinetic resolution of 2-phenoxymethyloxirane to (*S*)-phenoxymethyloxirane with 96.6% ee. Using the micelles reduced the amount of catalyst and the reaction time compared to the use of a homogenous catalyst. This enhanced catalytic performance was attributed to the hydrophobic microenvironment of the micelle core. Following reaction, the product was recovered by extraction with ethyl acetate. The polymer could be recovered from the aqueous phase by freeze drying. Upon reuse, the reaction times were increased [105]. Using a similar approach, asymmetric Rh ligands, ((2*S*,4*S*)-4-Di-phenylphosphino-2-(diphenylphosphinomethy)pyrrolidine moieties, were incorporated in the hydrophobic block of an amphiphilic block copolymer. A block poly (2-oxazoline) copolymer precursor containing ester groups in the hydrophobic block was synthesized, the esters were converted to free carboxylic acids, and the amino-functionalized ligands were coupled to the carboxylic acid groups on the polymer. The resulting polymer formed micelles in water at 0.9 mmol (1000-fold above its critical micelle concentration) and was complexed with Rhodium (I). The resulting catalytic micelles were used for enantioselective hydrogenation of (*Z*)-α-acetamidocinnamic acid to (*R*)-*N*-acetyl-phenylalanine. The product was extracted with ethyl acetate. Conversion was limited to 45–48%, which as was attributed to the high polarity of the substrate. Higher conversion and turnover frequencies were observed with more hydrophobic substrates [106]. 

Building on this approach, block copolymers with additional functional moieties (e.g., for crosslinking) have been integrated with functional handles for attachment to metal complexes. For example, using cationic ring-opening polymerization, Weck and colleagues synthesized an amphiphilic ABC-triblock copolymer based on poly(2-oxazoline). The block copolymer contained a hydrophobic block (A) and hydrophilic block (C); the middle block (B) was used for crosslinking. The terminal ester groups of the hydrophobic block were hydrolyzed and converted to carboxylic acids as functional handles to attach hydroxyl-functionalized Ru(II)-porphyrin complexes. The copolymer was complexed with Ru-porphyrin and micellized. The resulting micelles were crosslinked via thiol-yne chemistry with UV-radiation. The crosslinked micelle had a hydrodynamic diameter of 32 ± 6 nm, as determined by dynamic light scattering. The Rh-loaded micelles were used for epoxidation of styrene, with terminal alkenes as substrates in water, with H_2_O_2_ as the oxidant. For example, 1-hexene was converted to 1,2,-epoxyhexane with 99% conversion in 24 h, and vinylcyclohexane reacted to 94% conversion in 48 h. However, 2-bromostyrene only achieved 39% conversion within 48 h. The slow rate of reaction was attributed to electron withdrawal and steric hindrance near the reaction site. For reuse, micelles were recovered by ultrafiltration (10,000 cutoff membrane). When reused, conversion of styrene remained high (greater than 95%) for three reuses [107]. The block copolymer was also complexed with Co(II) acetate under an inert atmosphere and oxidized to produce Co(III) salen-loaded micelles (catalyst in the core). The resulting micelles had a hydrodynamic radius of 24 ± 6 nm, as determined by SEM, with 0.24% cobalt content (by ICP-MS). The micelles were used for hydrolytic kinetic resolution of terminal epoxides in water. Starting with epoxyhexane, the use of the micelles resulted in 52% conversion, with more than 99% ee within 15 h. However, fewer hydrophobic epoxides reacted minimally under the same conditions (e.g., conversion of epichlorohydrin was 5%). Thus, a one-pot competitive reaction between epoxyhexane and epichlorohydrin demonstrated a substrate in which selectivity was based on the hydrophobicity of the epoxides. Micelles were recovered by ultrafiltration with a 30,000 MWCO membrane and reused for as many as five cycles. Whereas minimal metal loss was observed by ICP-MS, the reaction rate decreased with each cycle of reuse. For additional reuses, the catalysts were reactivated with acetic acid to regenerate the Co(III) acetate catalyst [108]. 

Overall, polymer micelles are a versatile platform for performing organic reactions in water, complementary to other amphiphilic systems, such as surface-active ionic liquids and designer surfactants. Tools in polymer synthesis have facilitated the incorporation of a wide range of catalysts with significant tunability in terms of hydrophobic microenvironment (i.e., material) and size. Polymer systems have also facilitated stimuli-responsive systems to ease product recovery. 

## 6. Multistep, One-Pot Reactions

Micellar systems have proven a versatile platform for performing a wide range of reactions with many classes of catalysts using water as the bulk solvent. Lipshutz and colleagues have been especially prolific in demonstrating a wide array of organic chemistries possible using designer surfactants (recently reviewed [16]). Building on this foundation, synthetic sequences involving several steps have been developed. Such multistep reactions are an important class of reactions in modern organic synthesis, with applications in medicinal chemistry and agrochemistry. The ability to perform multistep reactions in combined processes could improve the efficiency of chemical processing. For example, telescoping multiple reactions can reduce solvent waste by limiting the volume of solvent, as well as eliminating purification processes. Thus, using micellar reaction media to perform multistep reactions in one pot is a promising approach to reduce solvent waste [17]. 

Traditional surfactants have been used for such multistep reactions. For example, the ability to perform Mannich reactions (synthesis of a β-aminoketone from an amine, enolizable ketone, and non-enolizabe aldehyde) using acid or base catalysts in micellar dispersions (e.g., Triton X-10, or SDS) has been well established. The desired product precipitates and can be recovered by filtration [45,109,110]. Using Brij-30 surfactant with Pd-based catalysts, pharmaceutical products, such as telmisartan and methylated diflunisal, were achieved with at least 70% yield using 500–1000 ppm. Stille coupling was combined with other reaction steps to achieve three-step reaction sequences in one pot. Specifically, a styrene derivative was produced by Stille coupling of vinyl bromide and 4-chlorophenyl stannate followed by reductive amination with N-Boc piperazine, a secondary amine (without isolation of the intermediate), followed by Boc deprotection. The desired product was purified by column chromatography, achieving an overall yield of 75%. Additionally, two Stille couplings could be performed in one pot without additional catalyst. The resulting biaryls were achieved with a yield of more than 70% [111]. Here, we review multistep one-pot reactions using micellar reaction media, including designer surfactants and block copolymer micelles. 

### 6.1. Cascade Reactions Involving TPGS-750-M and Multiple Catalysts

Due to the wide variety of reactions that have been performed using TPGS-750-M micelles, they have proven to be a useful platform for combining multiple reaction steps in a one-pot process. The range of reactions that can be combined in one-pot sequences are highlighted, building in complexity (i.e., length of reaction sequences).

The use of micellar media (TPGS-750-M) with Pd/C catalysts (4000 ppm Pd) has enabled multistep syntheses of pharmaceutical intermediates using one-pot reactions. Building on the ability to perform nitro-group reduction to amines using hydrogen, additional reactions could be undertaken in sequence. For example, an intermediate to pazopanib was synthesized by nitro-group reduction followed by S_N_Ar reaction. The resulting product was achieved with an 80% overall yield. Alternatively, an S_N_Ar reaction could be performed, followed by nitro reduction, to achieve an intermediate to imiquimod. The desired product was achieved with an 88% isolated yield. Nitro reduction was followed by reductive amination to make an intermediate for primaquine; the yield was 65%. In another example, 2-fluoro-4-nitroanisole was hydrogenated using a Pd/C catalyst in TPGS micelles, followed by acylation, to produce a precursor to 5′F-amodiaquine in quantitative yield. The product could be isolated by filtration. Following filtration, reuse of the micelles and catalyst was possible [112]. Using this approach with TPGS-750-M for S_N_Ar reactions, a one-pot, two-step reaction was also performed involving an initial S_N_Ar of reaction of 1-fluoro-2-nitrobenzene followed by NO_2_ reduction using Zn and NH_4_Cl. After two reaction steps, an overall yield of 86% was achieved [113].

One-pot reaction sequences can also combine catalysts. For example, using TPGS-750-M micelles, a copper-based catalyst, and polymethylhydrosiloxane as a stoichiometric hydride source, asymmetric ketone reductions were performed at 0–22 °C using toluene as a cosolvent. A one-pot, two step reaction was performed by following the asymmetric reduction of 2-acetyl-6-bromopyridine with Suzuki-Miyaura cross coupling of the resulting bromopyridine with 4-fluoro-2-methylphenylboronic acid using a Pd-based catalyst. The product was recovered by extraction with ethyl acetate and purified by flash chromatography. The yield was 78%, with 93% ee [114]. Alternatively, Ni-based catalysts in TPGS-750-M were used for C-S cross coupling of the intermediate to afford axitinib, an antitumor agent, in a two-step process. The isolated yield for both steps was 69%. The residual Ni was 9.8 ppm, which is below the FDA limit (<25 ppm). Telescoped reactions were performed on a gram scale [115,116]. Using nickel-based catalysts with TPGS-750-M micelles, reduction of bromocyclopropane was demonstrated. Double reduction was optimal with 5 equivalents of sodium borohydride, 1.5 equivalents of pyridine, and 20% THF. Such reactions could be combined in one-pot reactions. For example, the double reduction could be followed by Pd-catalyzed Suzuki-Miyaura cross coupling of 2-(2,2-dibromo-1-methylcyclopropyl)ethyl 4-bromobenzoate and 4-methoxy-2-methylphenylboronic acid. The resulting cyclopropane containing biaryl was recovered by extraction with ethyl acetate, filtration, and flash chromatography. An isolated yield of 86% was achieved [117]. Using commercially available Pd-based catalysts, allylic substitution reactions (“Tsuji–Trost couplings”) have also been performed in aqueous TPGS-750-M micelles (1000 ppm of commercially available catalyst). Reactions of (*Z*)-but-2-ene-1,4,diyl dibenzoate and dimedone were performed on a multi-gram scale (over 4 g). The product was recovered by extraction with ethyl acetate and purified by silica gel chromatography. Multistep, one-pot reactions were also demonstrated. Pd-catalyzed allylic substitution formation was followed by cobalt (salen)-catalyzed dehydrogenation to the *N*-allylated indole with 54% isolated yield [118].

Similarly, iron-based particles produced with small amounts of precious metals are versatile catalysts used in one-pot, multistep reactions alone and in combination with other catalysts. For example, iron-based particles with palladium (500 ppm) have been used for Sonogashira coupling (copper free). Such reactions could be performed in sequence in one pot by adding an additional alkyne to the reaction vessel without isolating the intermediate. The desired diyne product was achieved with an 80% isolated yield [119]. Selective nitro-group reductions could have also been performed using iron particles with ppm levels of palladium [120,121] in TPGS-750-M. Such capabilities have been used as the foundation of multistep, one-pot reactions [122]. For example, the resulting micelles could be used for coupling of 4-bromoanisole and naphthalene-1-boronic acid. The product was achieved with 95% isolated yield. One-pot sequential reactions could be performed using the micelles. Heteroaryl iodide with carbamate and trimethylsilyl (TMS) protecting groups were generated and then underwent cross coupling with alkenyl tetrafluoroborate salt. The TMS groups were removed, followed by *tert*-butoxycarbonyl (Boc) deprotection. Amination was performed with bromobenzene to achieve a 2,4,5-substituted pyrazol-3-one bioactive compound. The overall isolated yield was 68% [123]. Iron-based particles with small amounts of palladium (80 ppm) and nickel (1600 ppm) could be used for primary amine formation via reduction of nitroarenes. For example, chloroaniline was achieved via reduction of 4-nitrochlorobenzene, with 96% isolated yield in 15 min. The resulting primary amine could be further converted via S_N_Ar addition (with K_3_PO_4_), followed by Suzuki-Miyaura coupling (with Pd(OAc)_2_, SPhos, and triethylamine) in a one-pot reaction. An overall yield of the three-step reaction of 94% was achieved. One-pot nitro-group reduction followed by amine protection and Fischer indole synthesis (using *p*-toluenesulfonic acid) was also achieved using this approach with Fe/ppm Pd +Ni nanoparticles in TPGS-750-M micelles [124]. 

**Figure 6:**
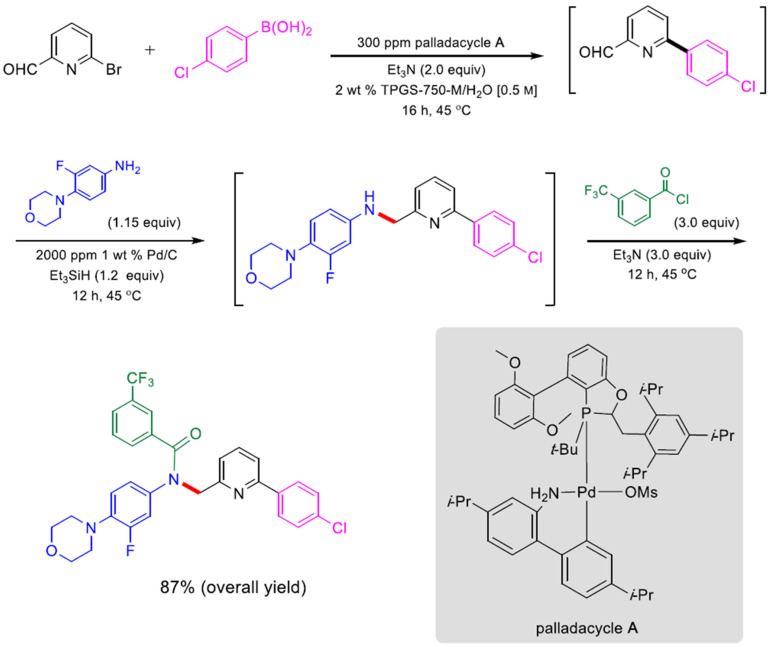
One-pot, three-step reaction sequence combining Pd-catalyzed Suzuki-Miyaura coupling with a reductive amination sequence, followed by acylation. Red indicates bond formation. The colors indicate how the reactants from each step were attached to form the final product. The product was then separated by extraction with a minimum amount of ethyl acetate and isolated by column chromatography. The desired product was achieved with an 87% yield in three steps. Adapted with permission from [114].

Versatile catalysts, such as palladacycles, have facilitated additional one-pot, three-step reactions. For example, reductive aminations have also been performed using Pd-based catalysts in TPGS-750-M micelles to produce pharmaceutical products, such as prozapine, cinacalcet, and fendiline, with ca. 90% yield. This capability could be combined to achieve tandem reactions in one pot. Specifically, Suzuki-Miyaura coupling was performed, followed by reductive amination and acylation (Figure 6). The product was recovered by extraction with ethyl acetate and purified by silica gel chromatography. An 87% yield of the desired product was achieved in three reaction steps. This class of catalyst also facilitated one-pot synthesis of celecoxib (a nonsteroidal anti-inflammatory agent) in a two-step method using Pd-based catalysts in TPGS-750-M micelles. Amination of bromosulfonamide was performed, followed by the addition of an unsymmetric β-diketone. The product was recovered by extraction with ethyl acetate and purified by column chromatography. The overall yield was 67% [114]. 

**Figure 7:**
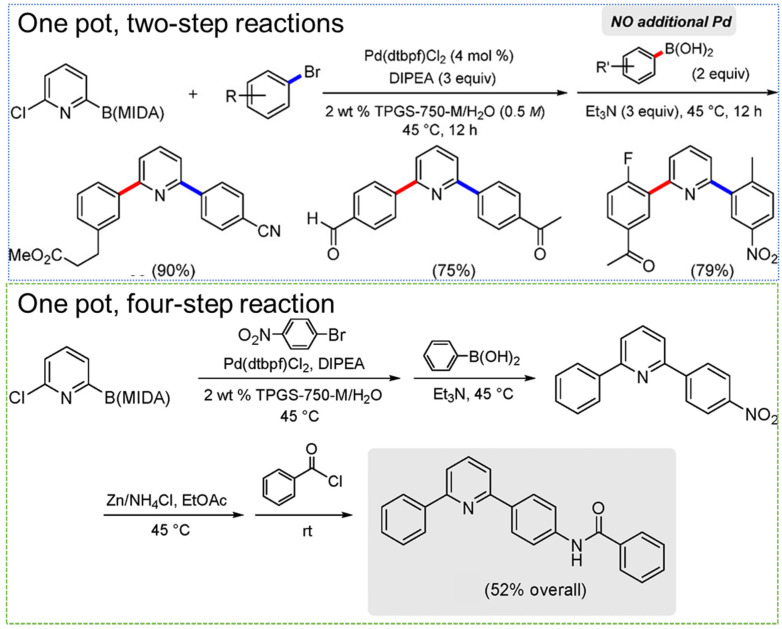
One-pot, two-step reactions to access to 2,6-disubstituted pyridines using micellar catalysis and Pd-catalyzed Suzuki-Miyaura cross-coupling reactions. Red and blue indicated bond formation in separate reaction steps. One-pot, four-step reaction to produce a drug analogue for Hedgehog signaling antagonist. Products were isolated by extraction with ethyl acetate and flash chromatography. Adapted from [125] with permission.

Building on these examples, longer sequences (e.g., 4 reaction steps) in one pot have been demonstrated. As a starting point, for Suzuki-Miyaura reactions, Pd(dtbpf)Cl_2_ was used in 2 wt% TPGS-750-M in water with DIPEA as the base. Typically, a 1:1 ratio of the two coupling partners is used at 45 °C. Following the coupling reaction, nickel-catalyzed hydrodehalogenation could be achieved by adding a nickel catalyst, ligand, base, and hydride source to the reaction. For example, 6-fluoro-2-B(MIDA)-pyridine could be coupled with an aryl bromide, followed by removal of the 6-halogen. Hydrodefluorination was achieved with 100% selectivity. The overall yield was 93%. As an alternative to dehalogenation, a second cross-coupling reaction was performed in one pot but without adding more palladium catalyst to the reaction vessel. By adding additional base and an arylboronic acid, a second coupling reaction was achieved to afford diarylated products, with overall yields of 75 to 90% (Figure 7). Building on these results, a four-step, one-pot reaction to produce a pharmaceutical analog to a hedgehog signaling antagonist was performed. Specifically, 6-chloro-2-B(MIDA)-pyridine was used as a starting material underwent two successive Suzuki-Miyaura cross-coupling reactions, followed (without intermediate isolation) by nitro-group reduction to the free amine by adding zinc and NH_4_Cl, with ethyl acetate as a cosolvent. For the fourth reaction, benzoyl chloride was added at room temperature to achieve the desired product. Following the reaction, additional ethyl acetate was added to extract the product. The resulting isolated yield was 52% following flash chromatography [125]. Similar reaction procedures were developed by Novartis [17]. 

**Figure 8:**
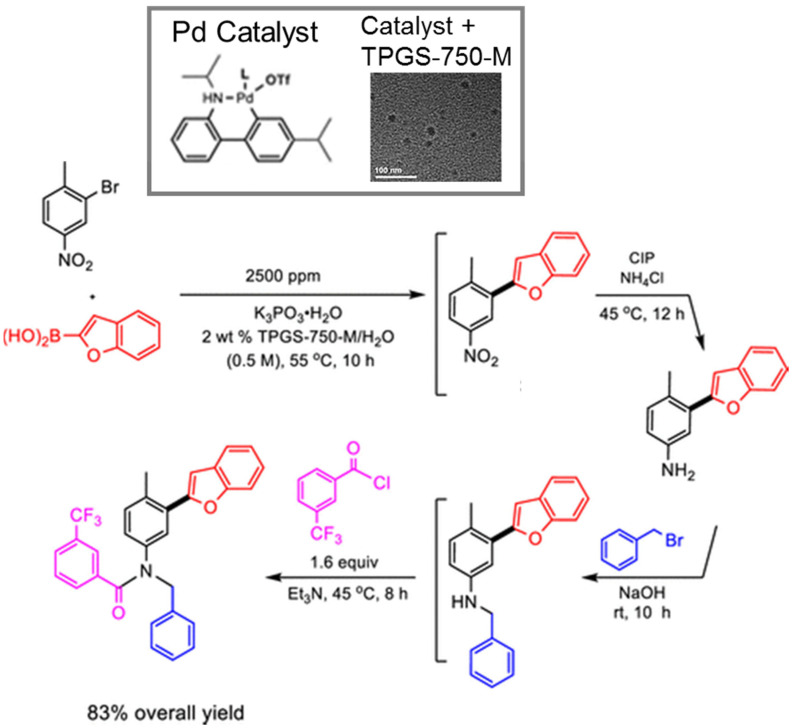
One-pot, four-step reaction sequence: (1) Suzuki-Miyaura coupling (Pd-catalyzed), (2) nitro-group reduction, (3) N-alkylation, and finally, (4) acylation in water using TPGS-750-M without isolation of intermediates. Bold indicates bond formation. The colors indicate how the reactants from each step were attached to form the final product. The final product was achieved with an 83% yield. The final product was isolated by extraction and flash chromatography. Adapted with permission from [126].

Another example of a one-pot, four-step reaction sequence was demonstrated using new biarylpalladacycles as precursors for Pd-based catalysts. These Pd-based catalysts were used for various types of reactions in TPGS-750-M dispersed in water, including Suzuki-Miyaura and Heck coupling. Stille cross couplings were demonstrated and used to produce a pharmaceutical product OSU 6162. The product was isolated by extraction with ethyl acetate, and the reaction was performed on a multi-gram scale with 87% isolated yield. For single-step reactions, the products were recovered by filtration (if solid) of decanting (if an oil). The yields were typically greater than 90%. Building on this capability, a one-pot, four-step reaction process was demonstrated without isolation of the intermediates. The representative process involved a Suzuki-Miyaura coupling, nitro-group reduction using carbonyl iron powder, N-alkylation, and acylation (Figure 8). The product was recovered by extraction with ethyl acetate and purified by flash column chromatography. The overall yield was 83% [126]. 

**Figure 9:**
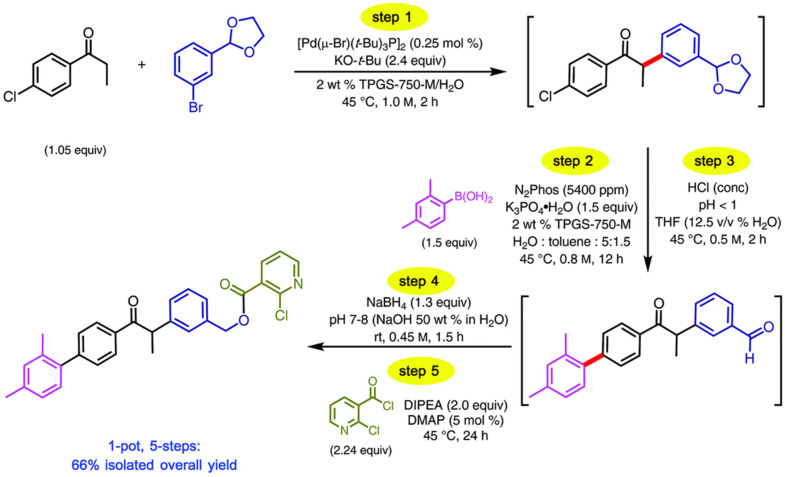
One-pot, five-step sequence starting with α-arylation of ketone using Pd-based catalysts in TPGS-750-M micelles. Red indicates bond formation. The colors indicate how the reactants from each step were attached to form the final product. The product was recovered by extraction with ethyl acetate and flash chromatography, with an overall isolated yield of 66%. Adapted from [127] with permission.

Further building in complexity, a five-step, one-pot reaction starting with α-arylation of 4′-chloropropiophenone with 1,3-dioxolane-containing aryl bromide was performed using Pd-based catalysts in TPGS-750-M micelles. Following isolation of the aryl chloride (66% isolated yield), Suzuki-Miyaura coupling was performed using a Pd catalyst with in situ ligand exchange. Without isolation of the intermediates, the aldehyde was generated, reduced to the benzylic alcohol, and treated with 2-chloronicotinonyl chloride to achieve the desired ester product (Figure 9). The product was isolated by extraction with ethyl acetate and further purified by flash chromatography, resulting in a 66% isolated yield [127].

### 6.2. Cascade Reactions Involving TPGS-750-M and Multiple Surfactants

**Figure 10:**
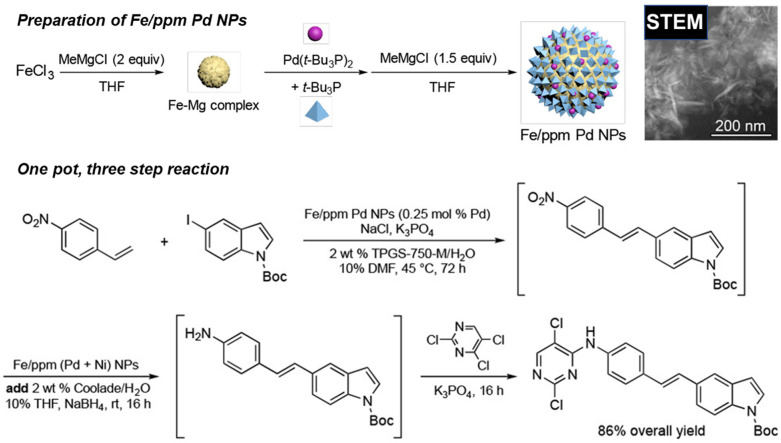
Preparation of iron-based particles containing ppm levels of palladium (Fe/ppm Pd). Initially formed NPs were spherical, measuring 2–5 nm. Upon addition of an aqueous solution of TPGS-750-M, the spheres were converted to nanorods (scanning TEM (STEM) image shown). A one-pot, three-step reaction sequence involving Mizoroki-Heck coupling was subsequently performed without isolation by nitro-group reduction using added Fe/ppm (Ni + Pd) NPs and ligandless nanoparticles in 2 wt% Coolade/H_2_O to avoid foaming from NaBH_4_. The resulting aniline was then treated directly with 2,4,5-trichloropyrimidine, leading to an S_N_Ar reaction. The final product was isolated by extraction and flash chromatography with 86% overall isolated yield. Adapted with permission from [128].

To achieve multiple reaction steps, combining multiple catalysts is often required. Combining multiple designer surfactants for different reaction steps is also possible. For example, TPGS-750-M was combined with Coolade, with antifoaming properties. In the first step, TPGS-750-M was used with iron-based particles containing ppm levels of palladium (Fe/ppm Pd) prepared using t-Bu3P as a ligand (Figure 10). The TPGS-750-M dispersion was used for Mizoroki-Heck coupling of 1-iodo-4-methoxybenzene and t-butyl acrylate with 78% yield of the cinnamate product. Yield could be increased to 95% by including excess phosphine ligand. The product precipitated as a solid and could be recovered by filtration. The reaction was scaled up to a gram scale with 87% yield. No residual Pd was detected in the product. Building on these results, sequential reactions were performed in one pot. For example, Mizoroki-Heck coupling of t-butyl 5-iodo-1H-indole-1-carboxylate and 1-nitro-4-vinylbenzene was followed (without isolation) by nitro-group reduction using Fe/ppm (Ni + Pd) NPs with Coolade (an antifoaming) surfactant. The resulting aniline was treated with 2,4,5-tricholorpyrimidine in an S_N_Ar reaction. The final product (*t*-butyl (*E*)-5-(4-(2,5-dichlorophenyl)aminostyryl)-1*H*-indole-1-carboylate) was achieved with an 86% isolated yield following extraction and purification by chromatography. Another sequence of reactions that can be performed is Mizoroki-Heck coupling of 1-bromo-4-iodobenzene and 1-(4-3-(trifluoromethyl)phenyl)piperazin-1-yl)prop-2-ene-1-one to produce a bromide product which can then be used (without isolation) in a Suzuki-Miyaura cross-coupling reaction with (1-methyl-1*H*-indol-5-yl)boronic acid. The biaryl product ((*E*)-3-(4-(1-methyl-1Hindol-5-yl)phenyl)-1-(4-(3-trifluoromethyl)phenylpiperazin-1-yl)prop-2-en-1one) was achieved with 82% overall yield following extraction and purification by chromatography. A one-pot, three-step reaction sequence involved Mizoroki-Heck coupling, followed, without isolation, by nitro-group reduction using added Fe/ppm (Ni + Pd) NPs and ligandless nanoparticles in 2 wt% Coolade/H_2_O to avoid foaming from NaBH_4_. The resulting aniline was then treated directly with 2,4,5-trichloropyrimidine, leading to an S_N_Ar reaction. The final product was isolated by extraction and flash chromatography with a 86% overall isolated yield (Figure 10) [128].

Reaction steps involving solvent-free conditions and micellar media have also been combined. Allylations of aryl/heteroaryl ketones were not possible in micellar media but could be performed without solvent. Reactions were performed with liquid and solid substrates. The reaction could be performed on a gram scale. The solvent-free reactions were telescoped with reactions performed in micellar media without isolation of the intermediates. Lipshutz and colleagues demonstrated a one pot, four-step reaction sequence starting with solvent-free ketone allylation, followed by ring-closing olefin metathesis, Suzuki-Miyaura coupling, and olefin hydrogenation (reduction). Without isolation of the intermediates between each step, the overall yield was 78% following purification by column chromatography [129].

### 6.3. Cascade Reactions to Produce Industrially Relevant Products 

**Figure 11:**
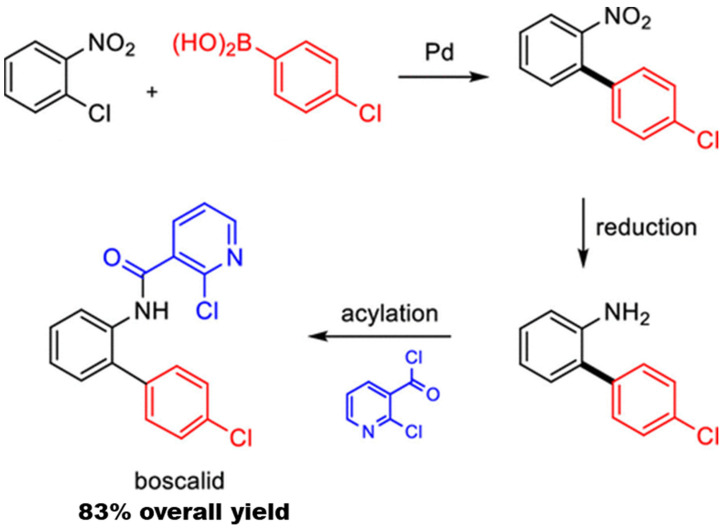
One-pot, three-step synthesis of boscalid (fungicide) involving Pd-catalyzed Suzuki-Miyaura cross coupling, followed (without isolation of the intermediates) by nitro-group reduction of the nitrobiaryl intermediate and amide formation using TPGS-750-M micelles. The product was isolated by extraction with ethyl acetate and further purified using flash chromatography. An 83% overall yield was achieved. Gram-scale (1.42 g) production was demonstrated. Adapted from [130] with permission.

Building on these capabilities (e.g., the ability to synthesize intermediates to pharmaceutical products), the ability to produce industrially relevant compounds leveraging one-pot, multiple step processes has also been demonstrated. For example, using TPGS-750-M, the fungicide boscalid has been achieved in a tandem, three-step reaction. Specifically, Pd-catalyzed Suzuki-Miyaura cross coupling was followed (without isolation of the intermediates) by nitro-group reduction of the nitrobiaryl intermediate and amide formation (Figure 11). The product was isolated by extraction with ethyl acetate and further purified using flash chromatography. After three reaction steps, the overall yield of boscalid was 83%. Gram-scale (1.42 g) production was demonstrated [17,130]. Additional examples of such syntheses using micellar reaction media are highlighted. 

The antimalarial drug pyronaridine has also been achieved using micellar reaction media. The initial reaction step was copper-catalyzed Ullmann coupling between 2,4-dichlorobenzoic acid and aminopyridine using TPGS-750-M. The intermediate was recovered by precipitation, filtration, and recrystallization. The use of the surfactant increased the yield (>99%). The reaction and product isolation were followed by cyclization/deoxychlorination using POCl_3_ in chloroform. The intermediate was precipitated with NH_4_OH (isolated yield, 87%). This reaction and product isolation were followed by an S_N_Ar reaction with *p*-aminophenol and a double Mannich-like reaction in TPGS-750-M in aqueous solution to yield pyronaridine. The desired pyronaridine product precipitated and was obtained in quantitative yield. The last two steps were performed in one pot. The overall sequence involved four individual reaction steps performed in three pots. The overall yield was 87% [131]. 

Traditional surfactants have also been used as reaction media for multistep synthesis of pharmaceutical products. For example, sonidegib, a Hedgehog pathway inhibitor developed for the treatment of basal cell carcinoma, has also been prepared using micellar catalysts containing ppm levels of Pd. The synthesis involved an S_N_Ar reaction using Brij-30 surfactant micelles followed by nitro-group reduction catalyzed by carbonyl iron powder, Suzuki-Miyaura cross coupling with triphenylphosphine with Pd(OAc)_2_, and amine-acid coupling. The five-step, three-pot reaction scheme was scaled up to a 10 mmol scale. Sonidegib was achieved with an 80% isolated yield following extraction with ethyl acetate and purification by column chromatography [132]. 

Flunarizine, a calcium channel blocker used to prevent migraines, has also been produced using micellar reaction media. Using SDOSS as a surfactant, 4,4′-difluorobenzophenone was reacted with NaBH_4_. The bis(4-fluorophenyl)methanol product was isolated by precipitation. The yield was 94%. Next, SOCl_3_ was added using the same micellar reaction media (10 wt% aq. SDOSS). The resulting chloro-bis(4-fluorophenyl)methane was isolated by extraction and column chromatography. A yield of 88% was achieved. Next, the resulting chloro-bis(4-fluorophenyl)methane was reacted with piperazine and t-BuOH using TPGS-750-M. The bis(4-fluorophenyl)methyl piperazine was isolated by extraction and column chromatography, with a yield of 81%. Ni-based catalysts in TPGS-750-M were used to react the bis(4-fluorophenyl)methyl piperazine with cinnamyl alcohol to produce flunarizine. The desired product was isolated by extraction and column chromatography, with a yield of 72%. Overall, the process required four reaction steps, all of which were performed using water as the bulk solvent, with an overall yield of 48% [133].

Lapatinib, a medication used to treat cancer, was produced in a five-step, three-pot method using micellar reaction media. The first step was an S_N_Ar reaction between 2-chloro-1-fluoro-4-nitrobenzene and (3-fluorophenyl)methanol using TPGS-750-M and KOH as a base. The intermediate product was isolated with a silica plug. The isolated yield was 98%. The resulting 2-chloro-1-((3-fluorobenzyl)oxy)-4-nitrobenzene was reduced using a Pd catalyst, TPGS-750-M, carbonyl iron powder, and NH_4_Cl. The intermediate product was recovered by filtration and extraction with ethyl acetate. The resulting intermediate was used in an S_N_Ar reaction with quinazoline in TPGS-750-M. The resulting heteroaryl iodide product was isolated by vacuum filtration. Next, Suzuki-Miyaura cross coupling with furanyl boronic acid was performed in the same reaction vessel using a Pd catalyst in ethanol. Without isolation, 2-(methylsulfonyl)ethan-1-amine hydrochloride and 2-picolineborane were added for reductive amination. The resulting lapatinib (free base) product was isolated by column chromatography (56% yield) or recrystallization in ethanol (57% yield). Typically, ca. 50 mg of product was achieved [134].

### 6.4. Other One-Pot Cascade Reaction Examples

The previous examples have generally used TPGS-750-M or traditional surfactants for at least one of the reaction steps. Additional one-pot, multistep reactions using other micelles systems, e.g., new designer surfactants and amphiphilic block copolymers, have been reported. For example, a new designer surfactant was developed: PS-750-M with a lipophilic tail linked to the nitrogen atom of a central proline unit further functionalized with an Me-PEG. This amphiphile was designed to mimic polar aprotic solvents, as the proline group introduced an increased degree of polarity into the micelle core [27], enabling transformations that traditionally require DMF, DMAc, NMP, and 1,4-dioxane. PS-750-M has been complexed with Pd and used as a surfactant and capping agent for Pd nanoparticles. The resulting nanoparticles dispersed in water were then used for cross coupling of carbene molecules. For example, N-tosylhydrazone was coupled with 1-iodo-4-methylbenzene as a model reaction at 60 °C for 24 h with 99% isolated yield. This cross-coupling reaction could be performed in tandem by first condensing the carbonyl and an *N*-tosylhydrazide to form the *N*-tosylhydrazone, then reacting with the aryl halide to achieve the final olefin. The final product was separated by extraction with ethyl acetate and further isolated by flash chromatography. Performing the multistep reaction in one-pot, the isolated yields were as high as 80% [135]. PS-750-M was also used to perform tandem reactions to produce α-azidoketone from styrene in a one-pot, multistep reaction. Notably, the conversion to the desired product was higher using PS-750-M micelles dispersed in water (at 60 °C under argon) than organic solvents, such as acetonitrile. Reactivity of the styrene in the micelle environment was attributed to in situ generation of hydrazoic acid and trapping of the radical intermediate. Subsequently, additional reactions could be performed. A one-pot carbonyl reduction of the α-azidoketone was performed to achieve α-azido alcohols followed by azide-alkyne cycloaddition (by adding CuI and alkyne) reactions to achieve β-keto triazoles. The product was isolated by extraction with ethyl acetate. Notably, the tandem reactions could be performed on a gram scale [136].

### 6.5. Polymer Micelles

In addition to traditional surfactants and designer surfactants, amphiphilic polymer micelles have also been used for one-pot, multistep reactions. For example, amphiphilic PEG-based micelles containing copper complexes have been used for tandem desilylation/Glaser reactions. Specifically, amphiphilic polyethylene-glycol-functionalized nitrogen ligands (i.e., PEG-functionalized 1,10-phenathroline and PEG-functionalized 2,2′-bipyridines) were synthesized. In aqueous media, the copper complexes aggregated in the core of the micelle surrounded by a PEG shell (spherical, ~100 nm in diameter). The self-assembled nanoreactors were then used for copper-catalyzed tandem desilylation/Glaser reactions using molecular oxygen in water. To perform a tandem desilylation/Glaser reaction, ethynylbenzene was reacted with 1,4-diphenylbuta-1,3-diyne in the presence of copper-loaded micelles. Notably, the yield was affected by the concentration of the amphiphilic ligand. Below the CMC, the yield was 40% or less. Above the CMC, the yield was 99%. Thus, the formation of micelles is important to the function of the catalytic activity of the ligand complex. Desilylation/homocoupling and desilylation/heterocoupling were also achieved. Symmetric products generally resulted in higher yields then asymmetric products (as much as 99% for symmetric products compared to 52–71% for asymmetric products). Homocoupling of terminal alkynes was also performed, with 63–71% yields. In this one-pot, multistep process, the product was isolated by cooling the reaction mixture to room temperature to cause the product to precipitate while the micelles remained soluble. The resulting mixture was filtered to recover the product (filter cake) and reuse the nanoreactors (filtrate). The copper-loaded micelles were reused directly as the solvent with the addition of ethynylbenzene. Following five reuses, the yield dropped from 97% to 89% [137].

Amphiphilic block copolymers have also been proven a versatile platform for performing one-pot, multistep reaction sequences. Tools in polymer synthesis have been leveraged to incorporate multiple catalysts into a single block copolymer. For example, triphenylphosphine-functionalized poly(2-oxazoline) block copolymer was synthesized. The resulting polymer had a hydrophilic block synthesized from 2-methyl-2-oxazoline. The hydrophobic block was a copolymer of 2-nonyl-2-oxazoline and 2-(5-amino-*tert*-butoxycarbonyl)-phenyl)-2-oxazoline. Following deprotection with methanolic hydrochloric acid to achieve free primary amine groups, the triphenylphosphine groups were linked to the hydrophobic block using carbodiimide coupling. The resulting polymer was complexed with rhodium and iridium and used for hydroaminomethylation of 1-octene, which is a three-step reaction sequence involving hydroformylation of an olefin, stoichiometric reaction with an amine, and hydrogenation to the desired amine. The reaction rates of the multiple reactions were balanced by varying the Rh/Ir ratio and the temperature to optimize conversion and selectivity. After 25 h at 130 °C and an Rh/I ratio of 2/1, the conversion of octene was 81%, and the selectivity for the desired amine was 69% [138].

In another example, amphiphilic poly(2-oxazoline) triblock copolymers were designed to site isolate two catalysts in different compartments of the same micelle. Micelles were achieved with carboxylic acid groups in the outer shell and tris(2-aminoethyl)amine (TREN base) groups in the micelle core. The resulting micelles were used for the two-step, one pot deacetalization–nitroaldol reaction of benzaldehyde dimethyl acetal to benzyl alcohol (acid catalyzed), followed by base-catalyzed Henry condensation. Using the dual-functionalized micelles, conversion was 99%, and the isolated yield of the desired product was 86%. Thus, compartmentalization of the incompatible catalysts facilitated the reaction sequence in a one-pot process using micellar reaction media [139].

Micelles combining organocatalysts and metal-based catalysts have also been developed. Weck and colleagues synthesized an amphiphilic triblock poly(2-oxazoline)-based copolymer containing two catalytic moieties; organocatalyst TEMPO was attached to the hydrophilic block of the micelle shell, and a rhodamine-based catalyst (rhodium *N*-tosylated 1,2-diphenyl-1,2-ethylenediamine (Rh-TsDPEN)) was incorporated into the micelle core via self-assembly. Although the catalysts were incompatible, they were site-isolated in different compartments of the micelle. The TEMPO-catalyzed oxidation of racemic secondary alcohols to ketone intermediates was followed by the Rh-TsDPEN-catalyzed asymmetric transfer hydrogenation to enantioenriched secondary alcohols. Using the micelles, the two-step reaction was performed in one pot. Using the reaction of 1-phenylethanol as a model substrate, conversion of 97% and 98% ee was achieved using the dual-functionalized micelles. The product was extracted from the micelles using diethyl ether, and the micelles were reused at least five times without a decrease in conversion [140]. In another example, amphiphilic block copolymers were functionalized with Rhodamine-based catalysts (Rh-TsDPEN) and coporphyrin catalysts. Upon self-assembly and cross linking, the coporphyrin was compartmentalized in the micelle core, and the Rh-TsDPEN catalyst was immobilized on the micelle shell. The resulting micelles were 36 ± 4 nm in hydrodynamic diameter, as measured by dynamic light scattering. The micelles catalyzed the hydration of phenylacetylene, followed by the asymmetric transfer hydrogenation to achieve a chiral alcohol. The product was extracted from the micelle solution using ethyl acetate, passed through a silica plug to remove the micelles, and analyzed by HPLC. The yield was 96% with 96% ee, demonstrating that the two incompatible reactions could be performed in one pot. Incorporating both catalysts within the same micelles reduced intramicellar diffusion compared to using two separate micelles (yield 60%) [141,142]. 

For a one-pot, three step reaction, a trifunctional micelle was used, consisting of an ABC triblock copolymer with carboxylic acid moieties in the hydrophilic block (micelle shell), Rh-based catalyst (Rh-TsDPEN) in the intermediate cross-linkable block (micelle middle layer), and 4-dimethyl-aminopyridine (DMAP) in the hydrophobic block (micelle core). Acid-catalyzed ketal hydrolysis of (1,1-dimethoxyethyl) benzene occurred in the micelle shell, followed by Rh-catalyzed asymmetric transfer hydrogenation and DMAP-catalyzed acetylation to enantio-enriched esters. The desired (*R*)-1-phenylethanol was obtained with 96% conversion and 97% ee. Using more hydrophobic ketals resulted in higher conversions. This result was attributed to affinity for the micelle core. Crosslinked micelles performed better than uncross-linked micelles, as indicated by the higher conversion. This difference was attributed to deactivation of DMAP by the acid in the uncross-linked micelles. Thus, the incompatibility of the catalysts in the uncross-linked micelles affected the performance more than crosslinking affected mass transfer of the substrate and product [143]. 

**Figure 12:**
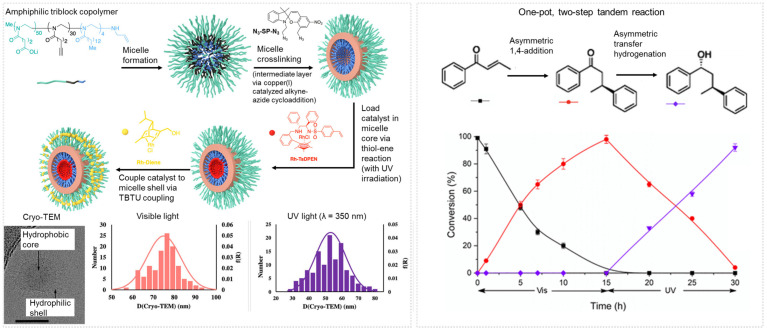
Amphiphilic triblock copolymers comprising a carboxylic acid salt-based hydrophilic block, a cross-linkable middle block containing a terminal alkyne, and a hydrophobic block containing an alkyl tail and a terminal allylamine for modification in the micellar core. Following micelle formation in water, the micelles were cross-linked using copper(I)-catalyzed alkyne-azide cycloaddition between the alkyne moiety and bifunctionalized spiropyran (N_3_-Sp-N_3_). A core-shell structure was observed by cryogenic TEM (cryo-TEM) (50 nm scale bar). The micelles were exposed to visible light (λ = 550 nm) for 15 min and to UV irradiation (λ = 350 nm) for 15 min. Upon irradiation with UV light, the diameter decreased from 70 nm to 58 nm (statistical size distribution is based on a sample size of at least 120 micelles). Two chiral Rh catalysts were immobilized in two separate microenvironments via orthogonal chemistries. First, the alkene functionalities in the core were reacted with a multivalent tetrathiol linker through thiol–ene click chemistry. A second hydroxy-functionalized Rh–diene complex was attached along the side chains of the shell via o-(benzotriazol-1-yl)-*N*,*N*,*N*′,*N*′-tetramethyluronium tetrafluoroborate (TBTU)/*N*,*N*-diisopropylethylamine (DIPEA)-mediated coupling. The reversible, photo-switchable behavior was retained after catalyst immobilizations. One-pot, two-step tandem reaction involving asymmetric 1,4 addition of phenylboronic acids to the *trans*-1-phenyl-2-buten-1-one reaction that occurs in the micelle shell was followed by Rh-TsDPEN-catalyzed ATH of (*S*)-1,3-diphenylbutan-1-one to yield the desired product, (1*R*,3*S*)-1,3-diphenyl-1-butanol, in the micelle core. The second reaction was triggered by UV light. Adapted with permission from [144].

Photoresponsive micelles have also been used for one-pot, multistep reactions. Polymer micelles were designed (based on multifunctional poly(2-oxazoline)s) to incorporate Rh-TsDPEN (1.63 per polymer chain by ICP-MS) in the micelle core and Rh-diene (1.83 per polymer chain per ICP-MS) in the shell upon self-assembly in water (Figure 12). The resulting micelles were approximately 74 nm in diameter, as measured by cryo-TEM. The micelles also contained a polymer block component that could be covalently cross-linked with spiropyran that created a photoinitiated gate due to the spiropyran to merocyanine transition. Upon exposure to UV light for 15 min, the micelle size was approximately 51 nm, as measured by cryo-TEM (Figure 12). This gate facilitated selective transport of substrates to direct the reaction pathway of two nonorthogonal enantio-selective transformations to produce secondary alcohol with two chiral centers. Specifically, the micelles were used for the one-pot, tandem reaction of phenylboronic acid to *trans*-1-phenyl-2-buten-1-one (Rh-catalyzed 1,4 addition) to (1*R*,3*S*)-1,3-diphenyl-1-butanol with 92% conversion and 99% ee (Rh-catalyzed asymmetric transfer hydrogenation). The reactions were typically incompatible because the first step requires KOH; however, the base deactivates Rh-TsDPEN, the catalyst for the second reaction. Typically, when performed in one pot, multiple intermediates and side products were observed. When the two catalysts were compartmentalized in the core and shell of the photo-responsive micelle, the two incompatible reactions took place in one pot by applying UV light after 15 h (complete consumption of the starting material). The photo-triggered change in the micelle initiated the asymmetric transfer hydrogenation to yield only the desired product (Figure 12). This result was attributed to selective transport of the substrates/reagents [144].

Overall, shell-crosslinked micelles based on amphiphilic triblock copolymers have been a useful platform for incorporating catalysts, as well as facilitating micelle recovery and reuse. Within a single self-assembled structure, compartmentalization of multiple, incompatible catalysts has been demonstrated. Such functionalized micelles have been used to achieve multistep reaction sequences in one pot. Thus, this is a promising platform, minimizing organic solvents. To date, the block copolymer micelle platform has been based on a single type of block copolymer. Scale up and the cost of block copolymer synthesis must also be carefully considered [97].

### 6.6. Novel Cascade Reaction Discovery Using Artificial Intelligence

Micellar reaction media have been a versatile platform for performing multistep reactions in one pot, with important applications medicinal chemistry and agrochemistry. Such a one-pot processing approach is promising for reducing solvent waste by limiting the volume of solvents, as well as eliminating purification processes. To date, processes involving one-pot, multistep reactions have been developed empirically. Discovery of new processes may be accelerated by machine learning tools [145]. Recently, there has been a growing body of work concerning automatic extraction of chemical reactions and experimental synthetic procedures from unstructured text using natural language processing [146,147]. The Conference and Labs of the Evaluation Forum in 2020/2022 related to Cheminformatics (CLEF ChEMU 2020/2022) Shared Task [148] released a dataset containing chemical reactions and their roles (e.g., solvent, starting material, reaction product, and catalyst). Entity recognition combined with relationship extraction facilitated identification of multiple reactions within a single patent. Recently, Guo et al. [149] demonstrated the ability to automatically identify reaction products from multistep reactions using sections of chemical journal articles. Vaucher et al. used natural language processing models (Pargraph2Actions) to predict experimental steps (add, stir, and extract) involved in chemical reactions from the Pistachio database (patents including the reaction SMILES strings with associated compound names and experimental procedures) [146]. The “Paragraph2Actions” model only accounts for linear sequences of actions [150]. Extending machine learning models to experimental procedures that involve multiple reaction steps would be valuable. Other remaining challenges include automatic extraction of chemicals included as images, including linking of the chemical term to the label within the text (e.g., “1A”). Ambiguity of the chemical name is also a challenge, as a single compound can have multiple synonyms (e.g., benzyl alcohol, phenyl methanol, (hydroxymethyl)benzene), benzylic alcohol, and phenyl methyl alcohol). Thus, normalization using a chemical dictionary would be an important step when using machine learning to automatically identify potential cascade reactions. 

## 7. One-Pot Chemoenzymatic Synthesis

Another class of one-pot reactions using micellar reaction media has leveraged enzyme-based catalysts. Enzyme-catalyzed reactions are performed in water under mild reaction conditions without organic solvents and result in high stereoselectivity [151]. Surfactant micelles can enhance the performance of enzyme catalysts by acting as a reservoir for substrates and products, decreasing noncompetitive enzyme inhibition [11]. Furthermore, using micellar reaction media, enzyme-catalyzed reactions can be combined in tandem with traditionally chemically catalyzed reactions. Ultimately, combining these capabilities broadens the complexity of products that can be achieved in one pot using micellar media. 

**Figure 13:**
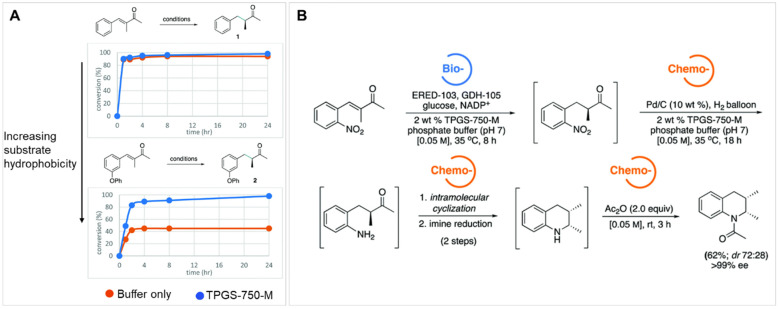
(**A**) Enone reductions with and without surfactant. Catalytic performance of the enzyme was not significantly affected by the presence of the surfactant, as evidenced by both the rate and the extent of conversion to product 1. However, reactions using substrates with increasing lipophilicity were affected by the surfactant. For product 2, conversion did not exceed 45% in phosphate buffer alone (0.1 M) at pH = 7. The addition of TPGS-750-M increased the conversion to 100% within the same 24 h time period. (**B**) Tandem four-step, one-pot process in TPGS-750-M micelles involving enzymatic enone reduction, nitro-group reduction, intramolecular cyclization/imine reduction, and acylation. The final product was isolated by extraction with 62% isolated yield. Adapted from [152] with permission.

For example, to achieve one-pot tandem processes combining chemical and biological transformations in water, an ene-reductase, enzyme-based catalyst was incorporated into TPGS-750-M micelles. The model substrate was 3-methyl-4-phenylbut-3-en-2-one. Complete conversion with 93% yield and with high enantiomeric excess (86% ee) was achieved. The presence of surfactant increased the conversion of substrates with increasing lipophilicity (Figure 13A). Multiple enzymatic steps could be performed in one pot. For example, a one-pot, biocatalyzed olefin reduction was followed by a keto-reductase-mediated reduction of the initially formed ketone. Building on these results, several one-pot, multistep chemo-/biocatalysis transformations were performed. For example, Pd-catalyzed Suzuki-Miyaura coupling was followed by ene-reductase-catalyzed reduction. A four-step reaction involved ene-reductase-catalyzed reduction, followed by Pd/C nitro-group reduction to the corresponding aniline and intramolecular cyclization and reduction to the corresponding imine, followed by a final Pd/C reduction to the dimethylated tetrahydroquinoline (Figure 13B). The final product was achieved with 62% overall yield and was isolated by extraction. No solvent was generated from workup of intermediates [151]. Alternatively, the chemically catalyzed reactions could be performed first followed by an enzymatically catalyzed reaction in one pot. For example, Pd-catalyzed dehydration of primary amines (4-bromobenzamide to 4-bromobenzonitrile) was performed, followed by (without isolation) Suzuki-Miyaura cross coupling with 4-acetylphenylboronic acid using Pd[dtbpf]Cl_2_ and (without isolation) an enzymatic reduction using TPGS-750-M in water as the reaction medium. The product was recovered by extraction, dried with silica, and further purified by column chromatography. A yield of the biaryl product of 96% with >99% ee was achieved [152]. Building on these capabilities, chemoenzymatic tandem processes to achieve more complex nonracemic products has been pursued. For example, Pd-catalyzed cross coupling to produce a ketone TPGS-750-M micelles was followed by enzymatic ketone reductions and subsequent enzymatically-catalyzed asymmetric reduction of the ketone-containing product to the corresponding nonracemic alcohols (≥99.5% ee). Three step, one-pot reactions were also achieved. Specifically, Rh-catalyzed 1,4-addition was followed by nitro-group reduction and enzymatic asymmetric ketone reduction. The one-pot reaction using TPGS-750-M resulted in 75% yield with 99% ee. The product was isolated by extraction and flash chromatography [11].

Other examples of chemoenzymatic, one-pot synthesis have been developed. Specifically, pig liver esterase, CALB lipase, and ω-transaminase were incorporated into TPGS-750-M micelles with metal-based catalysts (Pd-based for Suzuki-Miyaura cross coupling and Heck coupling or Ru-based catalysts for ring-closing metathesis of diene). Generally, the presence of the enzyme additives did not significantly impact the metal-based catalysts. Thus, one-pot reactions were performed. For example, Heck coupling was performed sequentially with enzymatic hydrolysis. Prior to adding the enzyme, the reaction mixture was neutralized and diluted. One-pot chemoenzymatic synthesis of 3-cyclopenten-1-ol was obtained by sequential ring-closing metathesis, followed by enzymatic hydrolysis of the ester group [153].

Lipase has also been used in combination with TPGS-750-M micelles to carry out various one-pot tandem chemoenzymatic reactions. Generally, lipase-catalyzed esterification reactions were performed in the presence of TPGS-750-M. The biocatalyzed reaction could be followed, without isolation, by chemocatalysis (e.g., Pd-catalyzed Suzuki-Miyaura coupling). As many as five steps could be performed sequentially in water, with overall yields of 65% [154].

Using polymer micelles, lipase has been combined with Cu(I)/bipyridine. Lipase is a well-established biocatalyst for ester hydrolysis and transesterification reactions. Cu(I)/bipyridine can be used for aerobic oxidations. Whereas both reactions can take place under moderate aerobic conditions, the ideal solvent for aerobic oxidation is acetonitrile, and the ideal solvent for lipase is aqueous media. To address this limitation, polymer micelles were used to compartmentalize the catalysts. The micelles comprised an amphiphilic block copolymer containing a cross-linkable hydrophobic block with bipyridine side chains to complex the copper catalyst. The resulting block copolymer formed micelles ca. 15–18 nm in diameter and was crosslinked with hexanediol dimethacrylate. The resulting micelles were used for oxidation of benzyl alcohol to benzaldehyde with 96% conversion and 94% isolated yield in 2 h at room temperature. The resulting micelles were also used in a one-pot reaction with lipase for a tandem reaction of benzyl acetate to benzyl alcohol by lipase, followed by oxidation to benzaldehyde by the Cu(I)-loaded micelles. The final product was isolated by extraction and purified by column chromatography. The overall isolated yield was 93% [142,155].

## 8. Process Scale Up

For translation of the discussed technological advances using micellar reaction media to industrial applications, the reaction scale is an important consideration. Generally, lab-scale reactions result in less than 100 mg of isolated product. To date, increasing the reaction scale has generally involved scale up of batch reactions to the gram scale. For example, using a traditional surfactant (Brij-30), cyanations using Pd-based catalysts with Zn(CN)_2_ as the cyanide source in the presence of polymethylhydrosiloxane (≥1 equivalent) could be performed on a gram scale. Specifically, pyrimidine was achieved with a 92% yield and isolated by extraction with ethyl acetate or filtration through a silica plug. Approximately 1.7 g of pyrimidine was produced. Such capabilities are an important class of reactions in the synthesis of drug intermediates. Importantly, the residual Pd was 2 ppm, which is lower than the FDA limit of 10 ppm [156].

Use of designer surfactants has also facilitated gram-scale reactions. For example, Lipshutz and colleagues demonstrated a 10 g scale using TPGS-750-M micellar reaction media and model reactions. The model reactions were a halogenated nitroaromatic reacting with a secondary amine, as well as a heteroaromatic polyhalide coupling with a nonracemic primary amine. Cosolvent (THF) was used to prevent the formed solid material from impeding stirring. With sufficient stirring, reactions were completed within 2 to 4.5 h at 45 °C. At the 10 g reaction scales, isolated yields of approximately 90% were achieved. Product isolation involved extraction with ethyl acetate and column chromatography. Alternative product isolation strategies were noted as an area for future improvement [157]. Hydrogenation of styrene could also be performed on a multigram scale using Pd/C catalysts and TPGS-750-M micelles with isolated yields ca. 80–90%. The product was recovered by distillation of the reaction mixture, so no solvent was required. One-pot, multistep reactions have also been achieved on larger scales by successively adding catalyst and performing multiple reaction cycles [158]. 

Chemoenzymatic reactions have also been performed on a large scale using micellar reaction media. Using enzyme-based catalysts, production of chiral 4-piperidinol has been demonstrated on a multikilogram scale. Using DMSO as a cosolvent (15% in water) with TPGS-750-M, the product precipitated as the reaction proceeded and could be recovered as a solid. A yield 85% with a purity of 97% was observed. Overall, a 3.5 kg reaction scale was achieved [11]. 

**Figure 14:**
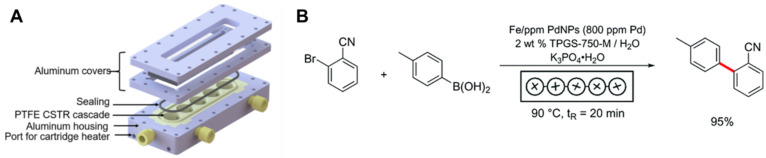
(**A**) Miniaturized CSTR cascade reactor. (**B**) Synthesis of a biaryl product in a continuous CSTR platform under flow conditions. Red indicates bond formed. The biaryl product is a key intermediate to sartan-containing pharmaceutical products, such as Losartan, to treat high blood pressure. Adapted with permission from [159].

In addition to scale up of batch processes, continuous processing using micellar reaction media has also been demonstrated using flow chemistry by cascading continuous stirred-tank reactors (CSTR) (Figure 14). Such reactors are reliable for performing solid-forming, multiphase reactions in continuous flow. The PTFE reactor block was comprised of five tanks (13 mm in diameter, 9 mm deep) connected by tubes (inner diameter, 2.5 mm) with an oscillator to enhance the transport of solids. Each tank was stirred by a magnetic stir bar. The entire reactor block was encased in aluminum housing for temperature control. The outlet stream was combined with 2-methyltetrahydrofuran using a T-mixer, followed by a static mixer to dissolve the solids formed during the reaction and prevent clogging of the reactor. As a model reaction, a stream containing Fe/ppm Pd NPs with 2 wt% TPGS-750-M/H_2_O was fed to the reactors with 4-bromoanisole and phenylboronic acid, as well as a stream of potassium phosphate. The reactors were heated to 90 °C. After reaching steady state (approximately three residence times), the product was isolated with 81% yield via extraction, followed by column chromatography. The use of this approach demonstrated continuous synthesis of a biaryl precursors to pharmaceutical products, such as sartans (blood pressure medications), Jakafi^®^ (a chemotherapy), and Zelboraf^®^ (a chemotherapy) [159]. Combining micellar reaction media with continuous-flow chemistry techniques, such as continuous extraction, may offer further opportunities to increase yield and minimize solvent use [160].

Alternatively, ultrafiltration has enabled continuous recovery of catalyst-containing micelles. Olefin epoxidation was performed using Triton X-100 micelles containing manganese porphyrins (Mn(TDCPP)Cl and Mn(TDCPPS_4_)Cl. Using hydrogen peroxide, propylene and 1-octene were epoxidized in the presence of imidazole. The turnover frequency of 1-octene was 250 h^−1^, and that of propylene was 49 h^−1^ at an initial pH of 8. The catalyst-containing micelles were recovered by ultrafiltration using a 3 kDa membrane. The flux approached 22 L/m^2^h (flux of water under the same conditions), and no catalyst was detected in the permeate [161].

These examples demonstrate the feasibility of continuous or large scales (kilogram) using micellar reaction media. The combination of chemoenzymatic, one-pot reactions using micellar reaction media has been especially valuable for large-scale production. The availability (or the complexity of the synthesis) of the designer surfactant or block copolymer may be an important consideration when scaling up reactions of interest. 

## 9. Process Innovations

Practically, in addition to reaction scale, the separation and isolation of the product as well, as reuse of the catalyst, are important considerations. The use of water as a solvent can enable catalyst recycling using biphasic extraction [162]. However, product extraction can be challenging due to the volume of the organic solvent used. For typical extraction processes, the volume of the organic solvent often exceeds the total volume of water used in the reaction by 30-fold. Furthermore, the resulting water would be contaminated with organic solvent and require purification. Additionally, the amphiphilic surfactant often forms emulsions, which can limit successful extraction of the product [7,15]. 

The use of designer surfactants has facilitated product isolation by “in-flask extraction” with a relatively small amount of solvent (e.g., ethyl acetate). When selecting a solvent for extraction, the distribution of the product between solvent and the aqueous phase is an important consideration. Ideally, the product would be highly soluble in the solvent, whereas the surfactant and catalyst would be insoluble. Such criteria would facilitate recovery and reuse of the surfactant and catalyst and prevent product contamination. The mutual solubility of water and the organic solvent is also an important consideration for solvent selection for extraction procedures in which the volume of the solvent is low. In addition to the solvation properties, the physiochemical properties must be also considered. For example, solvents with a low boiling point maybe desirable for recycling by distillation. Solvents with disparate densities relative to the aqueous phase would favor spontaneous phase separation, whereas two phases with similar densities may require centrifugation. It is also possible to predict the extraction efficiency and enrichment factor (a measure of purity) using a given based on partition coefficients calculated using solubility parameters [163]. 

Extraction is often combined with other separation techniques to achieve the desired product purity. For example, extraction can be performed following filtration [164]. Most commonly, extraction is followed by further purification using chromatography (flash chromatography or HPLC) [165]. Purification by column chromatography is solvent-intensive, with E factors of ~10,000–25,000 [166,167]. 

In some cases, extraction can be avoided. For products with limited solubility under the reaction conditions, filtration of the precipitated product has also been reported. If the selectivity is sufficient, filtration is the only step necessary to isolate the desired product. This approach has been demonstrated using enzyme-based catalysts in micellar reaction media (TPGS-750-M) [11]. The use of TPGS-750-M for Suzuki-Miyaura cross coupling is well established. With sufficient selectivity and reduced levels of side products and impurities, the product (API with biaryl nucleus) could be crystallized directly. Overall, initial crystallization of a salt followed by a second crystallization of the free form generated an API of sufficient quality [168]. 

**Figure 15:**
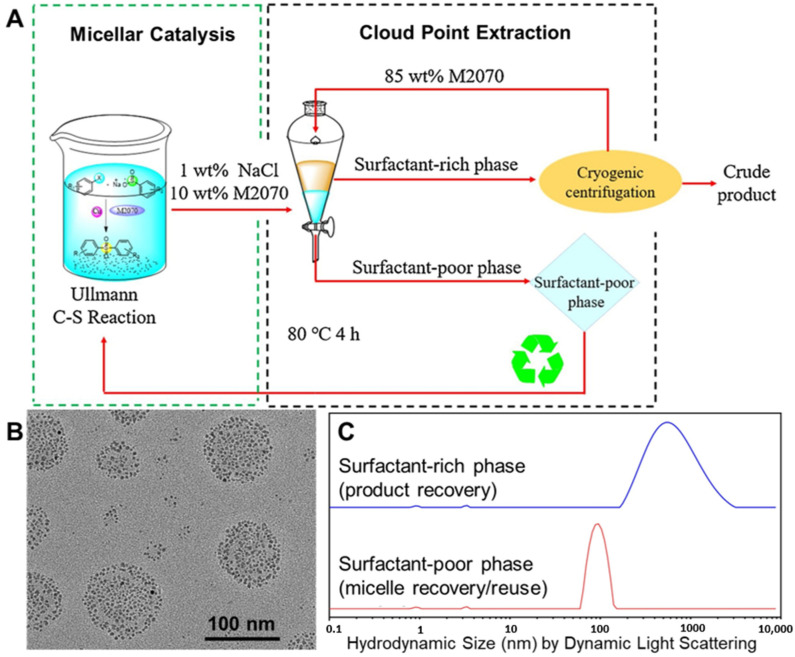
(**A**) Overview of a tandem process of micellar catalysis cloud-point extraction following copper-catalyzed Ullmann C-S coupling reactions in water using the nonionic surfactant amine-terminated polyether M2070. Following the reaction, the mixture was heated to above the apparent cloud point of M2070, resulting in phase separation. One “surfactant-rich” phase contained the product. The other “surfactant-poor” phase contained the surfactant-only micelles that could be reused. (**B**) TEM image of the M2070 aggregates at room temperature. Spherical aggregates ca. 4 nm in diameter further aggregated into large spheres ca. 110 nm in diameter. (**C**) Dynamic light scattering of the two phases after cloud-point extraction. The average micelle size was 93 nm in the surfactant-poor phase and 600 nm in the surfactant-rich phase containing product. Ultimately, the product was recovered as a solid via high-speed freezing centrifugation. Adapted with permission from [169].

For recovery of low-molecular-weight surfactants, cloud-point extractions have been demonstrated. M2070, a nonionic amine-terminated polyether, was used for Ullmann C-S coupling of sodium benzenesulfinate and *p*-iodoanisole using a copper salt catalyst. Yields of 90% could be achieved using CuBr. Following the reaction, the mixture was heated to 80 °C, which is above the apparent cloud-point temperature of the reaction mixture of ca. 72 °C. Upon heating, phase separation into two phases was observed. One “surfactant-rich” phase contained the product. The other “surfactant-poor” phase contained surfactant-only micelles that could be reused (Figure 15). To recover the product, the “surfactant-rich” phase was centrifuged. The crude product precipitated and was recovered by filtration and purified by silica gel chromatography. The extraction efficiency (amount of product in the surfactant-rich phase compared to the isolated yield) was more than 93% The surfactant-poor phase contained empty micelles and could be reused for reaction directly by adding more reagents [169].

To improve the recovery of micelles, combining magnetic nanoparticles with surfactants and block copolymers has shown promising results following various organic transformations in aqueous media (e.g., *C*-*C* coupling, acid-base catalysis, hydrogenation, oxidation, etc.). The nanoreactors (micelles and catalysts) can be recovered and reused. Use of magnetic separation is a promising approach to facilitate catalyst separation and reuse [170]. 

To date, significant progress has been made, demonstrating a variety of increasingly complex chemical syntheses using micellar reaction media. Such capabilities are especially promising for achieving products in the pharmaceutical industry. In addition to reaction scale, methods to isolate the product should be carefully considered. Techniques to fully recover the micelles without affecting catalytic performance (e.g., need to re-add additional catalyst after multiple cycles) would be particularly significant. Processes in which the product can be isolated with sufficient purity without solvent-intensive separation steps, i.e., chromatography and extraction, would be substantial in reducing the solvent waste associated with liquid-phase chemical processing using micellar media. 

## 10. Green Chemistry Metrics

One important goal of green chemistry is to reduce and prevent waste when manufacturing or using chemicals, particularly pharmaceutical products. The use of micellar reaction media has been useful for achieving such pharmaceutical products (e.g., [112,132,133,134]). To guide process design and provide benchmarks for future improvements, green chemistry metrics can be used. One metric for assessing waste is E factor, defined as:

(1) $$E-\mathrm{factor}=\frac{\mathrm{mass}\;\mathrm{of}\;\mathrm{waste}}{\mathrm{mass}\;\mathrm{of}\;\mathrm{prouct}}$$

When originally proposed, the calculation was intended for industrial processes to include “everything but the desired product”, including solvent and chemicals used in work up (a complete E factor, sometimes denoted as cEF) [3]. Ideal processes would have an E factor of zero, indicating zero waste. For multistep processes, all the unrecovered waste produced in every individual step would be divided by the mass of the final product, resulting from the entire synthesis. Adding E factors of the individual reaction steps results in arbitrarily low values. In practice, some limitations include idealized assumptions for solvent recycling, ambiguous inclusion of water, ambiguous system boundaries (e.g., simple vs. complete E factor), no inclusion of auxiliary chemicals that do not appear in the stoichiometric equation, and no explicit inclusion of energy requirements [171]. 

Generally, the E factor associated with using micellar reaction media has been ca. 3–7 when considering the solvents but no other auxiliary chemicals (e.g., K_3_PO_4_) [172,173,174]. When accounting for auxiliary chemicals, the E factor has been ca. 15 [175], comparable to other studies [176]. This reported metric has been most powerful for quantitatively comparing reactions performed in micellar reaction media compared to traditional reaction conditions using organic solvent [177,178]. For example, Feng et al. calculated an E factor for synthesis of aryl sulfides in the presence of phenyliodine(III) bis(trifluoroacetate). Using an organic solvent, the E factor was estimated to be more than 1100. Using TPGS-750-M, the E factor was approximately 13 (not including flash chromatography). The significant (orders of magnitude) reduction in E factor demonstrates the potential of micellar reaction media to reduce waste associated with liquid-phase chemical processing, particularly the solvent used for reaction. However, comparisons across reports are not always possible due to differences in system boundaries and inclusion of water or other auxiliary chemicals. Differentiating between E factor and solvent intensity (mass of solvent/mass of product) and explicitly defining the system boundary (i.e., the process steps involved to isolate the product) for the calculated metrics may identify potential areas for improvement.

Furthermore, for a more comprehensive assessment of green chemistry aspects, the E factor of the process should be considered in parallel with other metrics [179,180]. Process mass intensity (PMI) [181] and turnover frequency/turnover number (a measure of catalyst performance) have also been proposed as metrics to translate the Twelve Green Chemistry Principles into practice [182]. In practice, PMI is easier to calculate than E factor because it requires only knowledge about what is input into the reaction. PMI is defined as:

(2) $$\mathrm{PMI}=\;\frac{\mathrm{total}\;\mathrm{mass}\;\mathrm{used}\;\mathrm{in}\;a\;\mathrm{process}\;(\mathrm{kg})}{\mathrm{mass}\;\mathrm{of}\;\mathrm{final}\;\mathrm{product}\;(\mathrm{kg})}$$

Ideally, the PMI would be one; lower values indicate less waste [180]. As an alternative to E factor, the eco-scale metric has been proposed as a semi-quantitative tool. It is comprehensive and accounts for reagents, hazards, energy consumption, and waste. It may be an especially useful tool for laboratory-scale processes and evaluating new methodologies, such as one-pot, multistep reactions using micellar nanoreactors [183]. The green aspirational level (GAL) has also been proposed to account for synthetic complexity and maybe useful in the pharmaceutical industry [180,181]. Micellar reaction media have offered a versatile platform to reduce the use of solvents associated with a range of chemical reactions. To build on these capabilities, further considerations include “Green Chemistry Principle 6—Design for Energy Efficiency”, as purification and separation are the most energy-intensive chemical processes. Additionally, “Green Chemistry Principle 8—Reduce Derivatives”, i.e., avoiding inhibitors or protection groups, is an important consideration and could be quantified by atom economy [184]. For comprehensive process design, lifecycle analysis offers metrics and benchmarks for future improvements. It has been applied in bulk chemical products, and examples of its use in the pharmaceutical industry are emerging (e.g., using the GSK FLASC tool for life cycle analysis of synthetic chemistry). A more detailed description of such tools is available elsewhere [3]. 

Overall, green chemistry metrics may be a valuable tool for ensuring assessment of the sustainability of chemical processes implementing micellar reaction media. Practically, PMI is a comprehensive assessment of waste. The entire process (separation and purification of the product) should be carefully considered (e.g., [181]). Complementary analyses to consider additional factors, such as hazards and energy consumption, can also be evaluated. 

## 11. Outlook

Self-assembled micelle systems are reproducible and scalable. A wide variety of reactions have been performed using micellar reaction media. Because water is the bulk solvent, less solvent is used when compared to reactions performed in organic solvent. Furthermore, significant progress has been made, demonstrating a variety of increasingly complex, multistep chemical syntheses in one pot using micellar reaction media. However, recycling and recovery of materials with maintained catalytic performance remain a key challenge [13]. Whereas reuse of micellar systems for multiple cycles is commonly reported [162], catalyst deactivation can occur [103,108]. Additional catalyst may be needed for subsequent cycles [41,64]. Further characterization of metal leaching, product composition (rather than simply yield) and in-depth descriptions of isolation could enable gate-to-gate analysis, including energy consumption, reaction mass efficiency, and process economics [172].

Confinement of reactants, intermediates, and catalysts to micelles can enhance the efficiency of chemical transformations. If the reaction occurs in the hydrophobic micelle core, it is beneficial to increase the lipophilicity of all the reactants. Alternatively, if the reaction occurs at the hydrophobic–hydrophilic interface, the hydrophobicity of all the components needs to be fine-tuned to enhance apparent reaction kinetics. To date, this understanding for each system has been empirical. Increased use of computational tools to understand how the surfactant affects the performance of a given system (surfactant, catalyst, and reaction) could considerably accelerate this technology [62]. 

Additionally, to date, the majority of surfactants used have been derived from petroleum. Leveraging naturally derived surfactants and polymers may improve the sustainability of this approach. For example, sugar-based surfactants (glucose, lactose, or gluconolactone conjugated to amine-terminated polyether) have been used with Cu_2_O nanoparticles for C-S coupling reactions to produce zolimidine, a drug used to treat peptic ulcers, from commercially available materials with 64% isolated yield (purified by extraction and column chromatography). Minimal change in conversion was observed with as many as five reuses of the micelles (recovered by extraction) [185]. Glycan-based surfactants have also been used for nanoparticle synthesis. The resulting nanoparticle-loaded micelles may have promising applications for performing catalyzed reactions in water [186]. Naturally derived polymer, hydroxypropyl methyl cellulose (HPMC), is amphiphilic and can stabilize micelles [187,188]. At low concentrations (less than 0.2 wt% in water), HPMC can be used as an effective medium for organometallic catalyzed reactions. A wide range of reactions has been demonstrated, including (but not limited to) palladium-catalyzed amidation; Suzuki-Miyaura, Sonogashira, and Heck coupling; ruthenium-catalyzed cross-metathesis; and reductive aminations. The resulting products can be isolated via extraction with an organic solvent, such as methylene chloride or ethyl acetate. Product precipitation following the reaction has also been observed [187]. 

Overall, micelles are a versatile tool for performing organic chemistry in water. Polymer micelles are multifunctional and have facilitated stimuli-responsive (temperature, pH, and light) systems. Significant progress has been made in demonstrating multistep reactions in one pot. Moving toward process implementation, life cycle analysis should consider catalyst and surfactant selection, product isolation procedures, and the efficiency of catalyst and surfactant reuse in parallel with green chemistry metrics, such as the E factor. 

## Figures and Tables

**Table 1:** E factors, the ratio of the mass of waste to the mass of product, for various chemical industries. The ideal E factor is zero (zero waste); a higher E factor indicates more waste and an increased negative environmental impact [1,3].

Industry Segment	Production (tons/yr.)	E Factor	Total Annual Waste (tons)
Oil Refining	10^6^–10^8^	<0.1	10^7^
Bulk Chemicals	10^4^–10^6^	<1–5	10^6^
Fine Chemicals	10^2^–10^4^	5–50	10^5^
Pharmaceuticals	10–10^3^	25–100	10^5^

**Table 2:** Comparison of properties and micelle sizes of conventional surfactants and surfactants designed specifically for catalytic applications in water [55,56,58,59,60,61].

Surfactant	Charge	HLB	CMC (mmol)	Micelle Diameter (nm)
Conventional surfactants				
CTAB	Cationic	10	0.92	5–6
SDS	Anionic	40	7–10	4
Triton X100	Nonionic	13.5	0.23	7.5
Tween 20	Nonionic	16.7	0.059	8.5
“Designer” surfactants				
PTS	Nonionic	10	~0.1	~25
TPGS 750	Nonionic	13	~0.1	~50
Nok	Nonionic	10	~0.1	45–60
MC-1	Nonionic	15.8	Not reported	40–80

**Table 3:** Summary of reactions performed with various “designer” surfactants.

Surfactant	Key Surfactant/Micelle Properties	Sample Reactions
PTS	α-tocopherol (lipophilic), sebaic acid linker, PEG-600; spherical micelles ~25 nm in diameter	Pd-catalyzed cross coupling (Sonagashira, Suzuki-Miyaura, Heck), cross metathesis, ring-closing metathesis, amination, Negishi couplings, C-H activation, borylation, silylation, asymmetric hydrosilylation
TPGS 750	α-tocopherol (lipophilic), succinic acid linker, PEG-750; spherical micelles ~50 nm in diameter	Cross metathesis, Heck couplings, Suzuki-Miyaura couplings, Sonagarhira coupling, Stille couplings, amination, hydrogenation, Negishi couplings, reductive amination, S_N_Ar reactions, nitro-group reductions
Nok	β-sitosterol (lipophilic); MPEG-550 (hydrophilic); worm-like micelles 45–60 nm in diameter	Olefin metathesis, Pd-catalyzed cross couplings, aminations
MC-1	Sulfone component in lipophilic region of surfactant, 40–80 nm in diameter	Peptide synthesis
Coolade	Antifoaming, spherical micelles 30–40 nm in diameter	Nitro-group reductions
FI-750	Proline linker, spherical micelles 50–150 nm in diameter	Selective sulfonylation, cross couplings of quinoline and isoquinoline systems, α-arylation reaction of nitriles
PQS	Handle for attaching photocatalyst; size depends on attached catalyst	Ring-closing metathesis, cross metathesis, difunctionalization of alkenes, sulfonylation of enol acetates

## Data Availability

Not applicable.
